# JunD accentuates arecoline-induced disruption of tight junctions and promotes epithelial-to-mesenchymal transition by association with NEAT1 lncRNA

**DOI:** 10.18632/oncotarget.28026

**Published:** 2021-07-20

**Authors:** Subarna Ghosh, Priyanka Dey Talukdar, Abhinandan Bhattacharjee, Sarbani Giri, Nitai Pada Bhattacharyya, Urmi Chatterji

**Affiliations:** ^1^Cancer Research Laboratory, Department of Zoology, University of Calcutta, Kolkata 700019, West Bengal, India; ^2^Department of Otorhinolaryngology, Silchar Medical College, Silchar 788015, Assam, India; ^3^Department of Life Sciences, Assam University, Silchar 788011, Assam, India; ^4^Former Professor, Saha Institute of Nuclear Physics, Kolkata 700064, West Bengal, India; ^5^Centre for Research in Nanoscience and Nanotechnology, University of Calcutta, Kolkata 700098, West Bengal, India

**Keywords:** arecoline, tight junction, head and neck cancer, lncRNA-NEAT1, JunD

## Abstract

Head and neck cancers are highly prevalent in south-east Asia, primarily due to betel nut chewing. Arecoline, the primary alkaloid is highly carcinogenic; however its role in promoting tumorigenesis by disrupting junctional complexes and increasing risk of metastasis is not well delineated. Subsequently, the effects of low and high concentrations of arecoline on the stability of tight junctions and EMT induction were studied. A microarray analysis confirmed involvement of a MAPK component, JunD, in regulating tight junction-associated genes, specifically ZO-1. Results established that although arecoline-induced phosphorylation of JunD downregulated expression of ZO-1, JunD itself was modulated by the lncRNA-NEAT1 in presence of arecoline. Increased NEAT1 in tissues of HNSCC patients significantly correlated with poor disease prognosis. Here we show that NEAT1-JunD complex interacted with ZO-1 promoter in the nuclear compartment, downregulated expression of ZO-1 and destabilized tight junction assembly. Consequently, silencing NEAT1 in arecoline-exposed cells not only downregulated the expression of JunD and stabilized expression of ZO-1, but also reduced expression of the EMT markers, Slug and Snail, indicating its direct regulatory role in arecoline-mediated TJ disruption and disease progression.

## INTRODUCTION

In the past few years, there has been a drastic rise in the incidence of head and neck cancers worldwide [1]. Head and neck squamous cell carcinomas (HNSCCs) are highly prevalent in countries of south-east Asia, comprising 35–40% of all malignancies in India [2]. Cancer arising in the larynx is the most prevalent form of HNSCC (25–30%) and confers a negative effect on the quality of life [3, 4]. Habitual areca nut chewing, highly rife in Asian countries, is one of the potential causes of HNSCC [5]. International Agency for Research on Cancer (IARC) has declared the psychoactive areca nut to be carcinogenic to humans and chewing betel nut increases the risk of oropharyngeal cancer, independent of use of tobacco and alcohol [6]. The nut consists of various components, of which arecoline, the major alkaloid, is considered to be the most important carcinogen [7]. Salivary arecoline level in humans during betel nut chewing ranges from 5.66 to 97.39 μg/ml [8]. Arecoline induces cell proliferation, autophagy and enhances stemness property in various cancer models [9, 10]. In addition, reactive oxygen species (ROS) generation and epithelial-to-mesenchymal transition (EMT) have been implicated to the deleterious effects of arecoline. EMT, which is characterized by a loss of cell-to-cell contact, leads to repression of tight junction (TJ)-related proteins and eventually disruption of the TJs [11].

Multiple signaling pathways, such as MAPK/JNK and PI3K/AKT signaling pathways, are involved in the pathogenesis of HNSCC [12], and are indispensable for the growth and survival of cancer cells [13]. Cellular homeostasis and subsequent signaling are in turn regulated by cell adhesion molecules, which are severely disrupted in cancers [14]. Reports have linked activation of MAPK/JNK pathway to disruption of tight junctions (TJ) in various cell models [15, 16], resulting in reduced cell-to-cell interaction, loss of cell polarity and growth control, and eventually accentuating invasion and metastasis [17]. Studies have also demonstrated a correlation between reduced tight junction (TJ) components and tumor differentiation [18]. One such component, ZO-1, forms the backbone of the tight junctions in both epithelial and endothelial cells, and is indispensable for TJ assembly and its link to the actin cytoskeleton [19]. ZO-1 is unique among the TJ components as it organizes both structural and signaling components of the paracellular seal [20] and regulates a plethora of cellular activities, such as proliferation, differentiation, survival, and apoptosis [21].

One of the prominent effectors of MAPK/JNK activation is the AP-1 family of proteins, which are activated by various external stimuli and involved in cellular proliferation, differentiation and tumorigenesis. JunD, an AP-1 family member, plays a major role in cellular proliferation, anti-apoptosis, tumorigenesis, aggressive phenotypes and is regulated by phosphorylation of JNK [22]. JunD over expression has been associated with several cancer types [23, 24]. JunD knockout in mice increases levels of Bax, p53 and reduces levels of Bcl-2 [25]. JunD is involved in the induction of ROS production in prostate cancer [26]. JunD homodimers activates rat HSCs which contribute to the fibrogenic process through TIMP-1 activation [27]. A recent report has shown that JunD expression is positively correlated with precancerous and cancerous lesions in fresh oral tissues from different sites of oral cavity [28]. However there are no reports of the involvement of JunD in HNSCC. Therefore, whether arecoline-induced toxicity is mediated by JunD needs to be investigated. The present study attempts to understand the mechanism of arecoline-mediated carcinogenesis in acute and chronic chewers, which will facilitate in understanding the pathogenesis of the disease and development of more effective therapeutic strategies. Since MAPK pathway potentiates development and maintenance of HNSCC, the study also investigated the key players regulating the pathway. Here we report that JunD leads to down regulation of ZO-1 and abrogates tight junctions via activation of the JunD-NEAT1 axis in betel nut chewing HNSCC patients of India.

## RESULTS

### Dose-specific differential responses of arecoline in HNSCC cell lines

**Figure 1:**
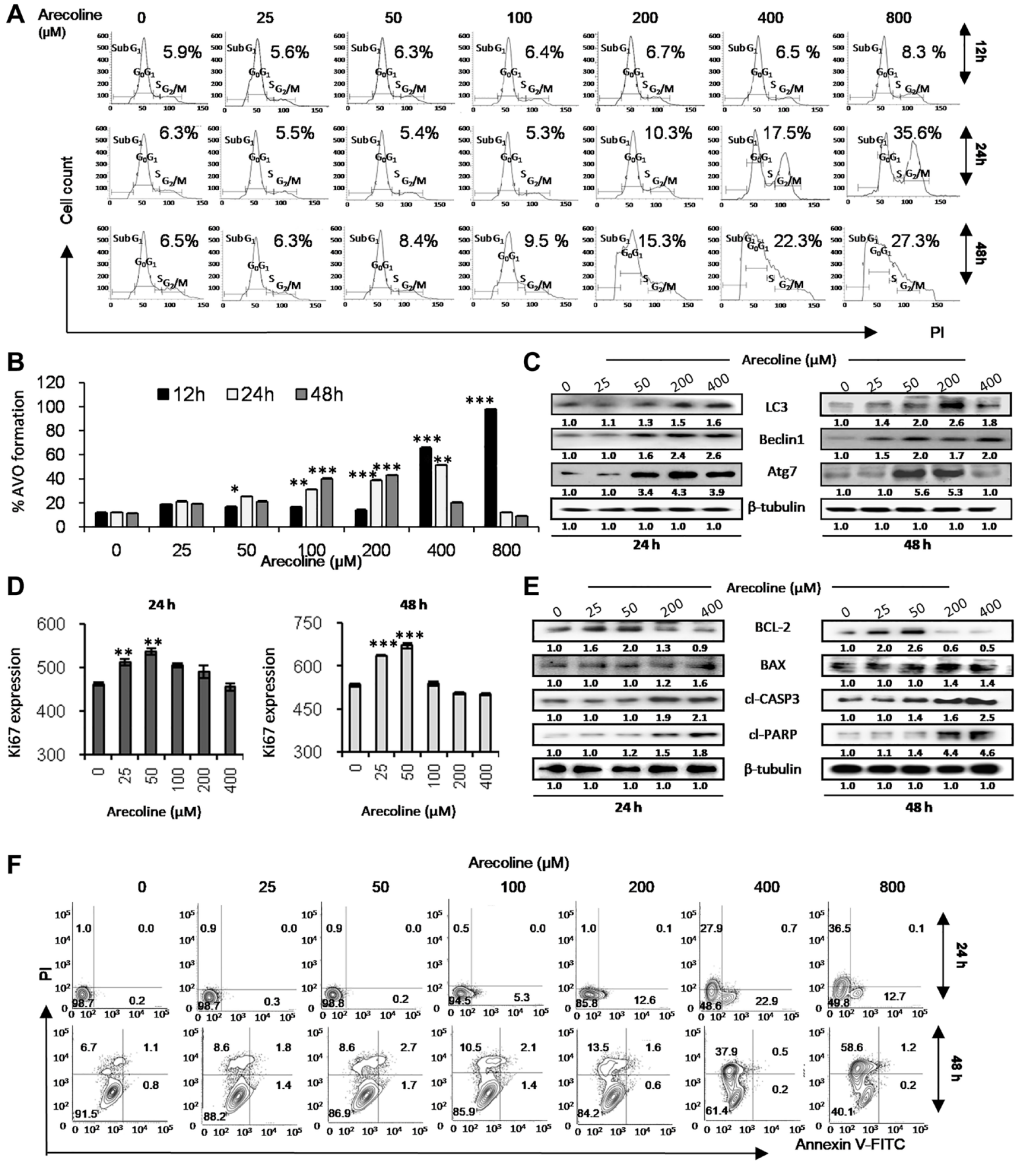
Arecoline induces apoptosis in HEp-2 cells at high concentrations and autophagy-mediated cell survival and proliferation at low concentrations. (**A**) Effect of increasing concentrations (0, 25, 50, 100, 200, 400 and 800 μM) of arecoline on cell cycle progression of propidium iodide (PI)-labeled HEp-2 cells after 12, 24 and 48 h of exposure as demonstrated by flow cytometry. The percentages indicate population of cells in G_2_/M phase of cell cycle. The graphical representation of the results is presented in Supplementary Figure 1D. (**B**) Effect of various concentrations of arecoline on induction of autophagy in HEp-2 cells after 12, 24 and 48 h of exposure, as indicated by increased formation of AVOs as compared to the respective control sets (no arecoline treatment). The fluorescent intensities indicated by PI fluorescence (x-axis) versus the number of cells (y-axis) graph are represented in Supplementary Figure 2A. (**C**) Western blot analysis showing increased expression of autophagy-related proteins Atg7, LC3-II and Beclin1 in HEp-2 cells upon treatment with arecoline in dose-dependent manner (0, 25, 50, 200, 400 μM) for 24 h (left panel) and 48 h (right panel). β-tubulin was used as the loading control. (**D**) Effect of arecoline treatment for 24 h (left panel) and 48 h (right panel) on HEp-2 cell proliferation as indicated by increased Ki-67. The fluorescent intensity of FITC was determined by flow cytometry and plotted in the semi-logarithmic graph of FITC fluorescence (x-axis) versus the number of cells (y-axis) (Supplementary Figure 2B). (**E**) Western blot analysis of apoptosis-related proteins such as Bcl-2, Bax, cleaved caspase 3 (cl-caspase 3) and cleaved PARP (cl-PARP) in HEp-2 cells upon treatment with different concentrations of arecoline for 24 h (left panel) and 48 h (right panel). β-tubulin was used as an internal control. (**F**) Flow cytometric analysis of cell death following arecoline treatment after 24 h and 48 h of using Annexin V-FITC/PI staining. FITC^-ve^/PI^-ve^ cells were designated as “live cells”, FITC^+ve^/PI^-ve^ as “early apoptotic cells”, FITC^+ve^/PI^+^ as “late apoptotic cells” and FITC^-ve^/PI^+ve^ as “necrotic cells”. The histogram is presented in Supplementary Figure 3C. All the experiments were performed three times. Each value is the mean ± S.D. of three different replicate experiments, each performed in triplicate. ^*^ *p* < 0.1, ^**^ *p* < 0.01, and ^***^ *p* < 0.001.

HPV^+^ laryngeal carcinoma cell line HEp-2 and HPV^−^ FOM (floor of mouth) tumor cell line SCC-131 was used to study the differential dose response of arecoline in cell lines with different HPV status. Analysis of dose response of HEp-2 cells to arecoline, performed at different time points, revealed that concentrations higher than 100 μM proved to be cytotoxic (Supplementary Figure 1A). Treatment with 200 μM arecoline for 48 h and treatment with 400 μM for 24 h reduced the viability of HEp-2 cells by 50%. Arecoline led to nuclear chromatin condensation in a dose- and time-dependent manner, indicative of cytotoxicity (Supplementary Figure 1B and 1C). In addition, the effect of arecoline on cell cycle progression was confirmed by flow cytometry (Figure 1A). Arecoline induced G_2_/M arrest at 400 μM and 800 μM and also significantly increased sub-G_1_ population when treated with 800 μM within 24 h of exposure. At 48 h exposure an increase in sub-G_1_ population can be detected in the 200, 400 and 800 μM concentrations (Supplementary Figure 1D). Concomitantly, neither MTT assay, nor proliferation and cell cycle analyses indicated significant toxicity in SCC-131 cells by arecoline at specified concentrations, compared to the HEp-2 cells (Supplementary Figure 1E) and hence, the HEp-2 cells were used for all subsequent studies.

A dose- and time-dependent increase in autophagy was observed, as indicated with autophagic vesicle formation detected by acridine orange and analyzed by flow cytometry (Figure 1B and Supplementary Figure 2A) and also by increased expression of LC3, Beclin1 and Atg7 proteins (Figure 1C and Supplementary Figure 5A). Flow analyses indicate increased autophagy at lower time points, which reduces with increase in time (Supplementary Figure 2A). Concomitantly, arecoline significantly up regulated the expression of Ki-67 at 25 μM and 50 μM concentration at 24 h and 48 h exposure, indicating survival and proliferation (Figure 1D and Supplementary Figure 2B). In addition, HEp-2 cells treated with arecoline for 24 h and 48 h showed a dose-dependent increase (*p* < 0.01) in Bcl-2 expression at lower concentrations (25 and 50 μM) which reduced significantly at higher concentrations (200 and 400 μM; *p* < 0.01). Conversely, expression of Bax, cleaved caspase 3 and cleaved PARP increased considerably after treatment with 200 and 400 μM arecoline (*p* < 0.01; Figure 1E and Supplementary Figure 5B). Annexin-PI assay by flow cytometry confirmed that after 24 h exposure and at concentrations higher than 200 μM there is increase in apoptotic, as well as necrotic, cell death (Figure 1F and Supplementary Figure 2C). However, at 48 h exposure, death is primarily due to necrosis (Supplementary Figure 2C). Since 50 μM and 400 μM displayed proliferative and apoptotic responses to arecoline, respectively, they were selected in subsequent experiments.

To determine whether arecoline-induced cell death was a result of ROS generation, HEp-2 cells were treated with various concentrations of arecoline for 24 h. Arecoline treatment significantly (*p* < 0.001) up regulated ROS generation in a dose-dependent manner, as indicated by fluorescence microscopy, where increased DCFDA staining was observed, and further quantified by flow cytometry (Supplementary Figure 3A). The status of antioxidant enzymes showed that arecoline significantly (*p* < 0.001) reduced the activity of antioxidant enzymes, CAT (400 μM), SOD (800 μM) and GSH (800 μM), when treated for 24 h (Supplementary Figure 3B). To further confirm that cell death induced by arecoline is directly an effect of ROS generation, cells were pre-treated with NAC, a ROS scavenger. Results indicated that pre-treatment with NAC significantly (*p* < 0.001) inhibited arecoline-induced ROS generation (Supplementary Figure 3C) and reduced arecoline-induced cell death (Supplementary Figure 3D).

### Arecoline induces EMT in HNSCC

**Figure 2:**
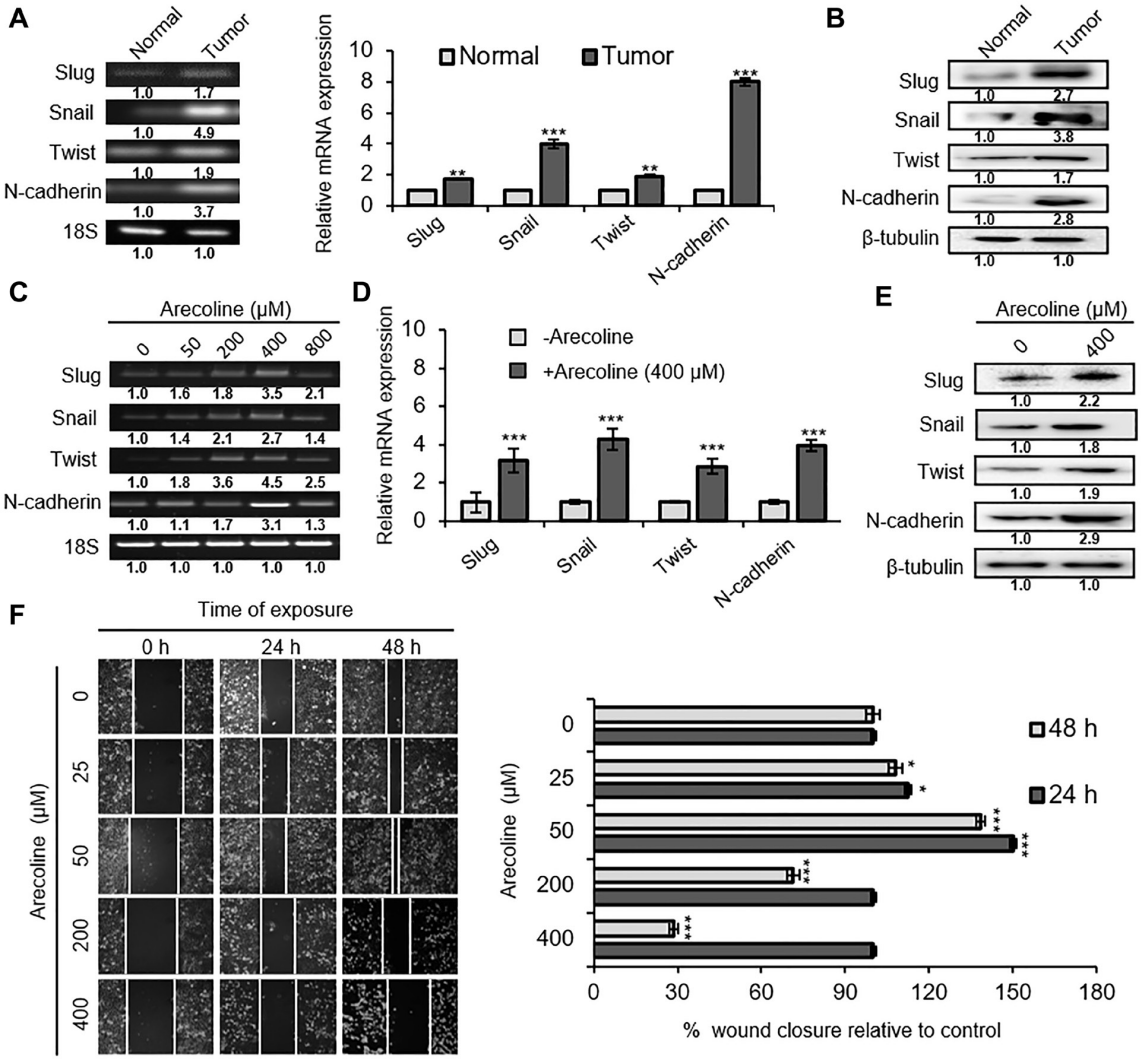
Arecoline induces EMT in a dose-dependent manner. (**A**) Expression of mRNA transcripts of EMT-related genes Snail, Slug, Twist and N-cadherin in oral tumor tissues and adjacent normal tissues of HNSCC patients, as evaluated by semi-qPCR (left panel) and qPCR (right panel). (**B**) Expressions of Snail, Slug, Twist and N-cadherin protein in oral tumor tissues and adjacent normal tissues of HNSCC cancer patients. β-tubulin was used as an internal control. (**C**) Dose-dependent mRNA expression pattern of the aforementioned EMT-related genes in HEp-2 cells following arecoline treatment for 24 h, as determined by semi-qPCR. (**D**) Expression of mRNA transcripts of EMT-related genes in untreated and arecoline-treated (400 μM for 24 h) HEp-2 cells. (**E**) Expression of Snail, Slug, Twist and N-cadherin protein in untreated and arecoline-treated (400 μM for 24 h) HEp-2 cells. β-tubulin was used as an internal control. (**F**) The phase contrast images representing the rate of migration of HEp-2 cells incubated in the absence and presence of different arecoline concentrations (25, 50, 200 and 400 μM) for 0 hr, 24 h and 48 h (left panel). The images were captured by using 20X objective lenses. The graphical representation of the same experiment is shown on the right. All mRNA expressions were normalized using 18S rRNA as the internal control. Each value is the mean ± S.D. of three replicate experiments, each performed in triplicate. ^*^ *p* < 0.1, ^**^ *p* < 0.01, and ^***^ *p* < 0.001.

Since quantity of arecoline differentially controlled proliferation versus death of HEp-2 cells, its contribution on epithelial-to-mesenchymal transition (EMT) was assessed. Expression of EMT markers, such as Slug, Snail1, Twist1 and N-cadherin was found to be significantly higher in HNSCC tissues compared to adjacent normal tissues, both at the mRNA transcript (Figure 2A; *p* < 0.001) and protein levels (Figure 2B; *p* < 0.01). To simulate the conditions, HEp-2 cells were treated with different concentrations of arecoline for 24 h. In accordance with the tissue scenario, arecoline treatment led to dose-dependent increase in the expressions of Slug, Snail1, Twist1 and N-cadherin transcripts, specially at 400 μM (Figure 2C; *p* < 0.001). Quantitative PCR (qPCR) analysis indicated up regulation of Slug (3.2-fold), Snail1 (4.3-fold), Twist1 (2.3-fold) and N-cadherin (4-fold) in arecoline-exposed cells (Figure 2D). The above results were further established at the protein level (Figure 2E and Supplementary Figure 5C). In addition, bidirectional wound healing assay was performed to confirm EMT induced by arecoline. Interestingly, wound closure after 24 and 48 h was more pronounced in 25- and 50 μM-treated sets but relatively retarded in 200- and 400 μM-treated sets, compared to the control (Figure 2F).

### Arecoline down regulates tight junction (TJ)-associated proteins in HNSCC

**Figure 3:**
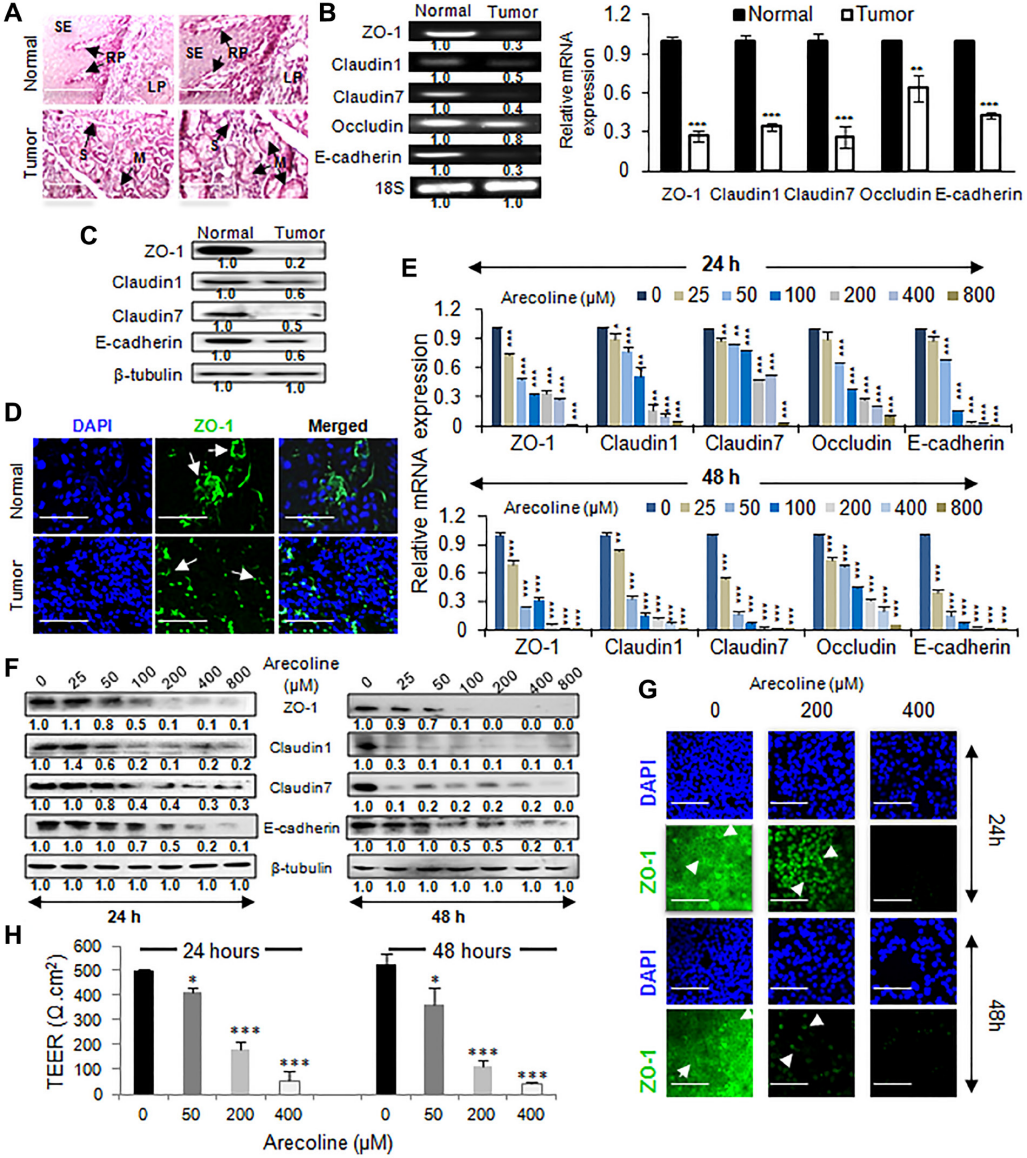
Arecoline disrupts tissue integrity and downregulates the expression of tight junction proteins in HNSCC. (**A**) Hematoxylin and eosin staining of tissue sections from normal and HNSCC patients showing disrupted tissue organization in tumor tissues. Arrows indicate lamina propria (LP), organized squamous epithelium (SE), rete pegs/ridges (RP), serous glands (S) and mucous glands (M). Scale: 200 μm. (**B**) The semi-qPCR (left panel) and qPCR (right panel) of expression of tight junction-associated genes in tumors (*n* = 5) and adjacent normal tissues (*n* = 5). (**C**) Alterations in expression of tight junction-associated proteins in normal and oral tumor tissues. (**D**) Immunofluorescence micrographs of tumor tissue and adjacent normal tissue of HNSCC patients, stained with DAPI and FITC-conjugated anti-ZO-1 antibody. The arrows indicate continuous membrane staining in normal tissue and punctuate staining of ZO-1 in tumor sections. Scale bar: 100 μm. Effect of arecoline on transcripts of tight junction-associated genes (**E**) and proteins (**F**) in HEp-2 cells upon treatment with various concentrations of arecoline for 24 h (left panel) and 48 h (right panel). (**G**) Immunocytochemical analysis for ZO-1 expression in HEp-2 after 24 h (left panel) and 48 h (right panel) incubation. The arrows indicate membrane staining of ZO-1 in control cells and cytoplasmic accumulation in arecoline treated cells. Scale bar: 100 μm. 18S rRNA was used as internal control for the PCRs and the evaluated mRNA expressions were normalized using 18S rRNA. β-tubulin was used as an internal control for western blots. (**H**) TEER analysis to determine integrity of tight junctions in cell monolyers in response to arecoline treatment at 0, 50, 200 & 400 μM doses for 24 h and 48 h. All the experiments were performed thrice. Each value is the mean ± S.D. of three different replicate experiments, each performed in triplicate. ^*^ *p* < 0.1, ^**^ *p* < 0.01, and ^***^ *p* < 0.001.

Since EMT entails deregulation of cell junctions and disruption of cellular architecture, tumor and adjacent normal tissues from cancer patients were stained to visualize the tissue architecture. Where normal tissue sections demonstrated a definitive tissue organization, tumor sections showed loss of cellular organization, severe epithelial dysplasia and hyperplasia of cells adjoining the mucous and serous glands (Figure 3A). Simultaneously, expression of TJ markers in tumor and normal squamous epithelial tissues revealed from both semiq-RTPCR and qPCR significant (*p* < 0.001) down regulation of ZO-1 (3.7-fold), CLDN-1 (2.9-fold), CLDN-7 (3.8-fold), OCLN (1.6-fold) and E-cadherin (2.3-fold) transcripts (Figure 3B). Concomitantly, protein expressions of the above TJ-associated markers were significantly (*p* < 0.001) down regulated in tumor tissues compared to normal tissues (Figure 3C and Supplementary Figure 6A). Immunofluorescence studies further supported disruption of ZO-1expression in the epithelial processes of the tumor tissues (Figure 3D and Supplementary Figure 6B). To ascertain the effects of arecoline on the expression of TJ markers, HEp-2 cells were treated with varying concentrations of arecoline for 24 and 48 h. It was observed that arecoline significantly (*p* < 0.001) reduced mRNA transcripts (Figure 3E) of the markers. Interestingly, expression of ZO-1, CLDN-1, CLDN-7 and E-cadherin protein did not change significantly at lower concentrations and at 24 h exposure periods, but at concentrations higher than 100 μM, significant reduction in protein expression was apparent. However, exposure for 48 h led to significant downregulation (*p* < 0.001) of the proteins even at the lower concentrations (Figure 3F and Supplementary Figure 6C). Immunocytochemical staining for ZO-1 confirmed dose-dependent loss of staining and redistribution of ZO-1 from the cell membrane to the cytoplasm (Figure 3G and Supplementary Figure 6D). In addition, experiments were performed to detect changes of TEER values as indicators of tight junction integrity in monolayers of HEp-2 cells. The TEER value of HEp-2 cells after 72 h was calculated to be ~500 Ω.cm^2^, indicating development of tight junctions and sound monolayer integrity. TEER was subsequently measured at specific times (24 and 48 h) after treatment with different concentrations of arecoline (50, 200 and 400 μM). After treatment of HEp-2 cell monolayers with arecoline, TEER decreased to almost 90% of the initial value (*p* < 0.001; Figure 3H).

### Arecoline augments stemness acquisition in HNSCC

**Figure 4:**
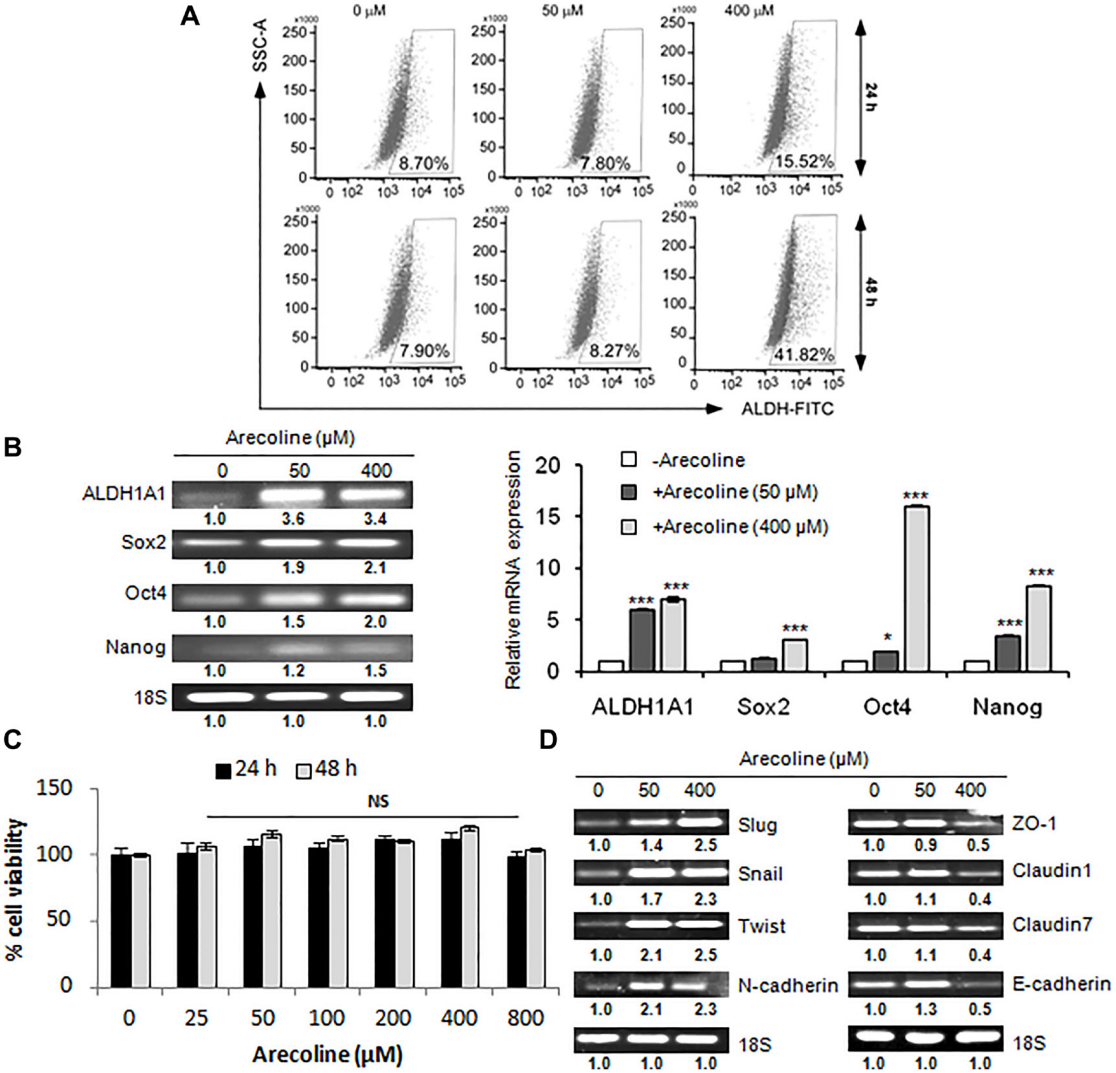
Arecoline augments stemness acquisition in HNSCC. (**A**) Flow cytometry analyses depicting enhancement of ALDH^+^ cells upon treatment of HEp-2 cells with arecoline (0, 50, 400 μM) for 24 h (upper panel) and 48 h (lower panel). (**B**) Expression of mRNA transcripts of stemness-related genes by semi-qPCR (left panel) and qPCR (right panel) in HEp-2 cells treated with arecoline for 24 h. (**C**) Percentage cell viability of HEp-2 spheroids upon treatment with different concentrations (0, 25, 50, 100, 200, 400, 800 μM) of arecoline for 24 and 48 h, as evaluated by MTT assay, indicating no significant toxicity. (**D**) mRNA expression of both EMT-related genes (left panel) and TJ-associated genes (right panel) of HEp-2 spheroids treated with 0, 50 and 400 μM arecoline for 24 h. 18S rRNA expression was used as internal control. All the experiments were performed thrice. Each value is the mean ± S.D. of three different replicate experiments, each performed in triplicate. ^*^ *p* < 0.1 and ^***^ *p* < 0.001.

Cancer stem cells (CSCs) are primarily responsible for growth, invasion and metastasis of HNSCCs [29]. Consequently, we assessed the status of CSCs in response to arecoline. Aldefluor assays of HEp-2 cells confirmed that arecoline considerably enriched the population of stem cells at 400 μM at 24 h (*p* < 0.01) and at 48 h (*p* < 0.001; Figure 4A). Expressions of CSC-associated markers were found to be significantly (*p* < 0.001) up regulated when treated with arecoline at the transcription level (Figure 4B). Effect of arecoline specifically on orospheres showed that arecoline did not have any cytotoxic effect on the CSCs (Figure 4C). Furthermore, spheroids treated with 50 μM and 400 μM arecoline for 48 h demonstrated enhanced expression of EMT markers (*p* < 0.001) concomitant with reduced expression of TJ-associated genes, especially after treatment with 400 μm arecoline (*p* < 0.001; Figure 4D).

### MAPK pathway mediators involved in TJ regulation by arecoline

**Figure 5:**
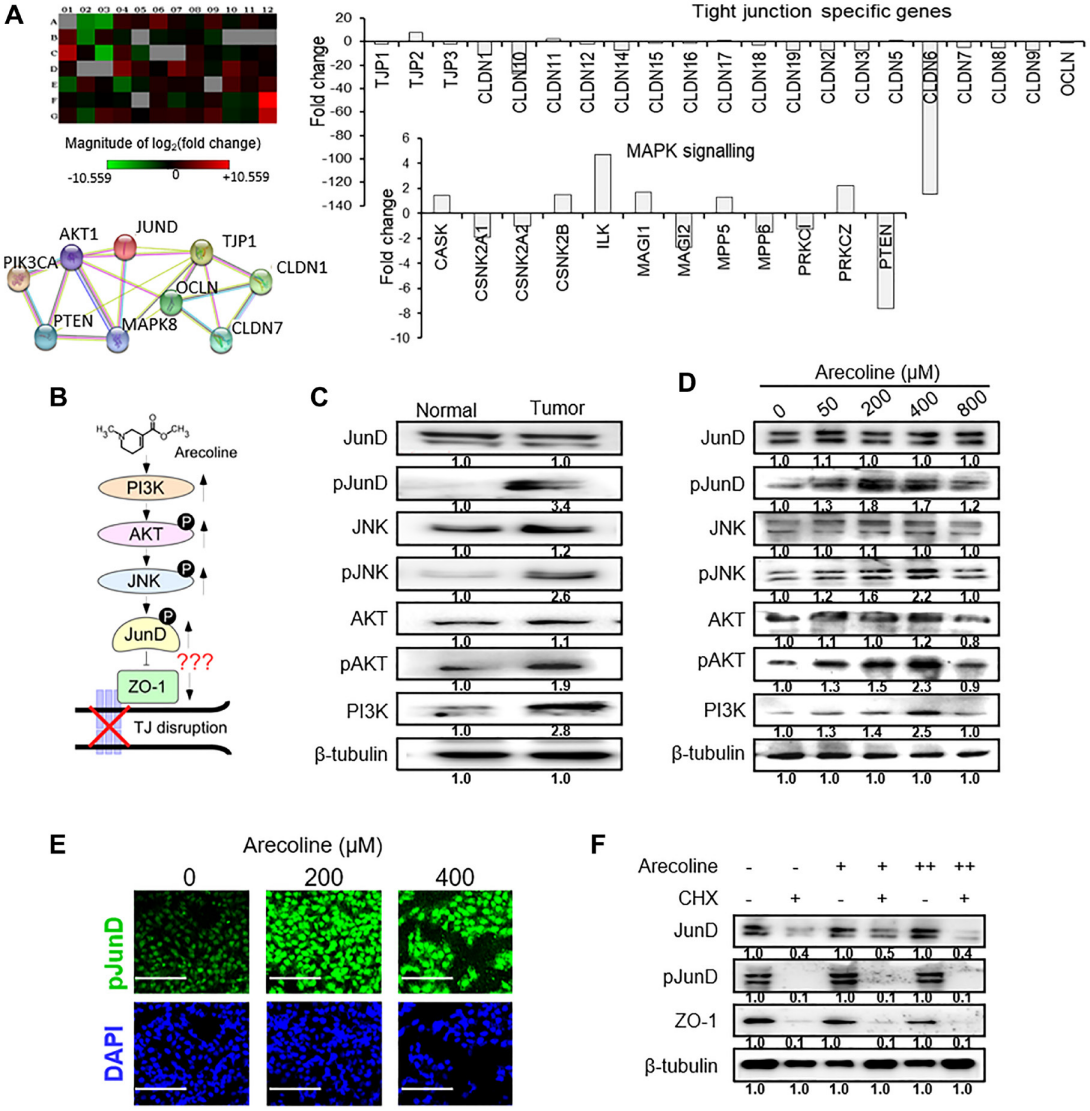
Arecoline-induced activation of the MAPK pathway mediators. (**A**) Microarray analysis (representative of 3 independent experiments) of tissue biopsy samples shows differentially regulated genes in tumor as compare to adjoining normal tissues of the same patient. Upper left panel depicts the heat map. Upper right panel represents differentially expressed genes related to tight junction and adhesion molecules. Lower right panel depicts expression pattern of G-protein and protein kinase signaling molecules. Lower left panel shows string analysis of interaction of candidate genes of the protein kinase signaling pathway and adhesion molecules. (**B**) Schematic representation of the plausible pathway components, PI3K/AKT/JNK/JunD, steering the inhibitory effects of arecoline on ZO-1. (**C**) Expression of MAPK pathway regulator proteins in oral tumor tissues (*n* = 3) and adjacent normal tissues (*n* = 3). The blot is representative of 3 different paired tissues. (**D**) Protein expression of MAPK pathway regulators in HEp-2 cells in response to different concentrations of arecoline treatment for 24 h. (**E**) Immunofluorescence micrographs of HEp-2 cells treated with arecoline for 24 h and stained with anti-pJunD antibody. DAPI was used to stain the nuclei. Scale bar: 100 μm. (**F**) Expression of JunD, pJunD and ZO-1 in HEp-2 cells incubated with 50 μM (+) and 400 μM (++) arecoline in the absence (–) and presence (+) of 100 μg/ml cycloheximide (CHX). β-tubulin was used as an internal control for all western blot analyses. All the experiments were performed three times and each value is the mean ± S.D. of three different replicate experiments.

To delineate the underlying molecular mechanism responsible for arecoline-induced disruption of tight junctions, a microarray analysis was carried out to identify the genes involved. It was apparent that the expression of TJ-associated genes was mostly lower in tumors than normal tissues (Figure 5A). Of the few genes that were significantly down regulated was PTEN (7.6 fold) (Figure 5A), which persuaded us to investigate components of the MAPK pathway in tumor and normal tissues. Data mining revealed putative regulatory molecules which may control expression of ZO-1, the primary TJ-associated molecule, based on which a possible regulatory pathway was formulated (Figure 5B). One possible candidate which may negatively regulate ZO-1 was JunD, a member of the transcription factor activator protein (AP)-1 family. Interestingly, significant increase (*p* < 0.001) in expressions of PI3K, phospho-JNK (pJNK), phospho-AKT (pAKT) and phospho-JunD (pJunD) were observed in the tumor tissues compared to normal tissues (Figure 5C; Supplementary Figure 7A). To confirm this finding *in vitro*, HEp-2 cells were treated with 50, 200, 400 and 800 μM of arecoline for 24 h and subjected to western blot analysis. As expected, a dose-dependent increase in PI3K, pJNK, pAKT and pJunD was noted (Figure 5D and Supplementary Figure 7B). Immunofluorescence micrographs of HEp-2 cells treated with arecoline also indicated enhanced pJunD (Figure 5E and Supplementary Figure 7C). To ascertain whether enhanced expression of JunD/pJunD was a *de novo* function or a question of protein stabilization in the presence of arecoline, HEp-2 cells were treated without or with 100 μM cycloheximide, an inhibitor of translation. Since in the presence of cycloheximide, protein expression was significantly reduced, it was apparent that arecoline led to *de novo* synthesis of JunD in the absence of cycloheximide (Figure 5F and Supplementary Figure 7D).

### Arecoline upregulates pJunD and mediates TJ disruption and cell motility

**Figure 6:**
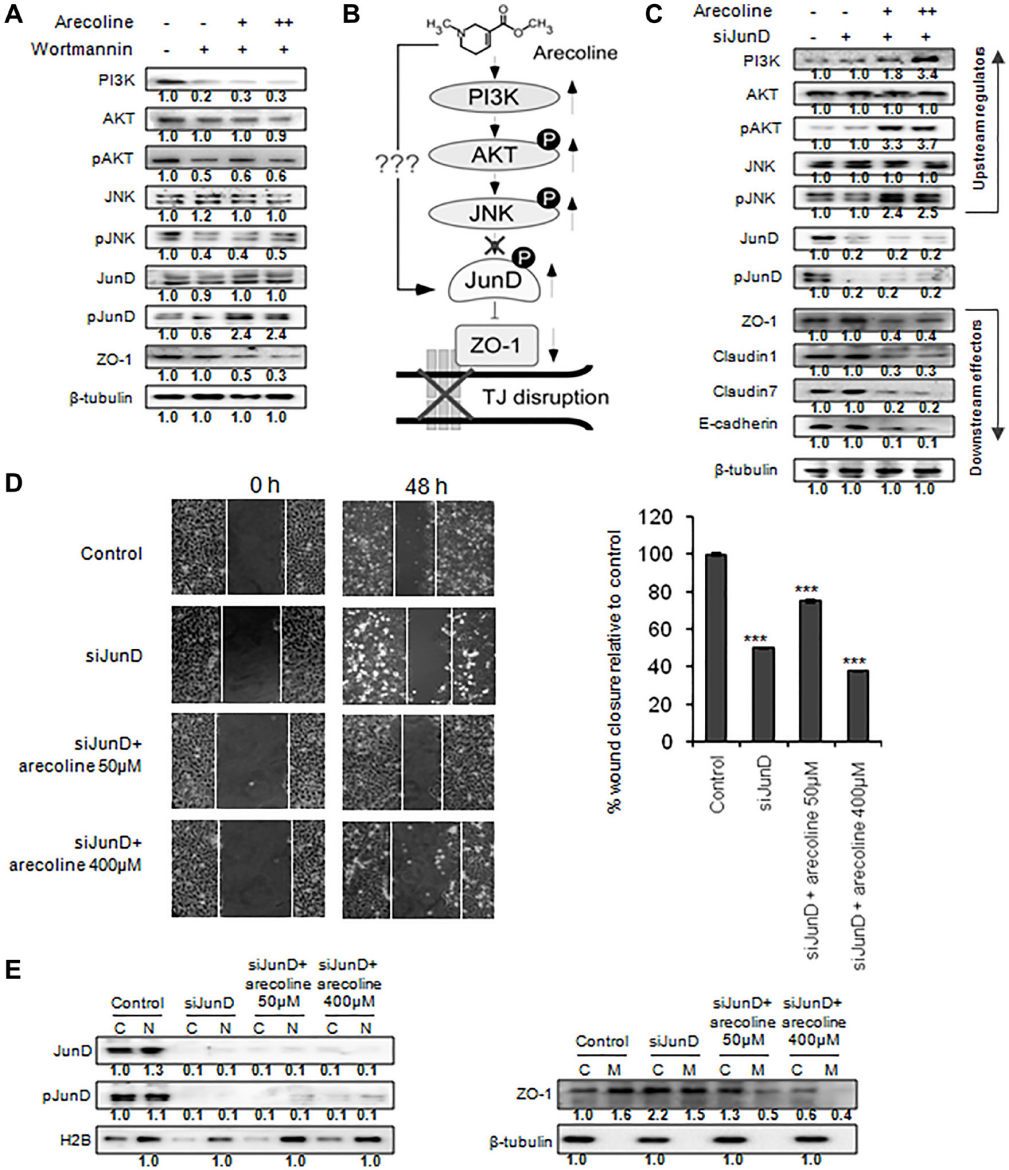
Arecoline mediates tight junction disruption by JunD phosphorylation and ZO-1 down regulation. (**A**) Differential protein expression of the MAPK pathway regulators in HEp-2 cells in response to 50 μM (+) or 100 μM (++) arecoline and 100 μM wortmannin. (**B**) Presumptive upstream pathway components orchestrating the inhibitory effect of arecoline on ZO-1, leading to tight junction disruption. (**C**) Effect of silencing JunD on MAPK and tight junction components in HEp-2 cells in absence and presence of 50 μM (+) and 100 μM (++) arecoline. (**D**) Phase-contrast images of bidirectional wound healing assay illustrating the effects of silencing JunD on migration of HEp-2 cells in absence and presence of arecoline after 48 h of incubation. Graphical representations indicate % wound closure. ^***^ *p* < 0.001 (**E**) Effect of JunD silencing followed by arecoline treatment on JunD, pJunD and ZO-1 in the cytoplasmic (C), nuclear (N) and membrane (M) fractions y. β-tubulin was used as an internal control for all western blots. All the experiments were performed thrice. Each value is the mean ± S.D. of three different replicate experiments.

To ratify the pathway proposed in Figure 5B, and ascertain that increased phosphorylation of JunD by upstream components led to down regulation of ZO-1, HEp-2 cells were treated with 50 μM and 400 μM arecoline in the presence of wortmannin, a specific inhibitor of PI3K. Figure 6A confirmed that inhibition of PI3K reduced phosphorylation of JNK, AKT and JunD, in the absence of arecoline. On the contrary, arecoline significantly increased phosphorylation of JunD (*p* < 0.01) in contrast to pJNK and pAKT, even in the presence of wortmannin (Figure 6A and Supplementary Figure 8A). This confirmed that in the presence of arecoline, PI3K, AKT and JNK did not directly affect JunD, and arecoline possibly affected the expression of JunD via an alternative mechanism (Figure 6B). Further, silencing JunD in HEp-2 cells clearly indicated reduction in expression of JunD and pJunD, concomitant with increased expression of ZO-1, which stabilized even in the presence of arecoline (Figure 6C and Supplementary Figure 8B). This phenomenon was not observed when scrambled sequences were used for silencing JunD (Supplementary Figure 4). However, alterations in expression of PI3K, pJNK, and pAKT in the presence of arecoline were independent of JunD regulation (Figure 6C and Supplementary Figure 4), confirming them as the upstream components of JunD. Effect of JunD on cell motility was assessed by bidirectional wound healing assay and it was found that silencing JunD retarded migration of HEp-2 cells significantly even in the presence of arecoline (*p* < 0.05; Figure 6D). Interestingly, silencing JunD sequestered ZO-1 proteins from the membrane to the cytosol, where they were stabilized even in the presence of arecoline (Figure 6E and Supplementary Figure 8C).

### NEAT1 lncRNA as an activator of JunD

**Figure 7:**
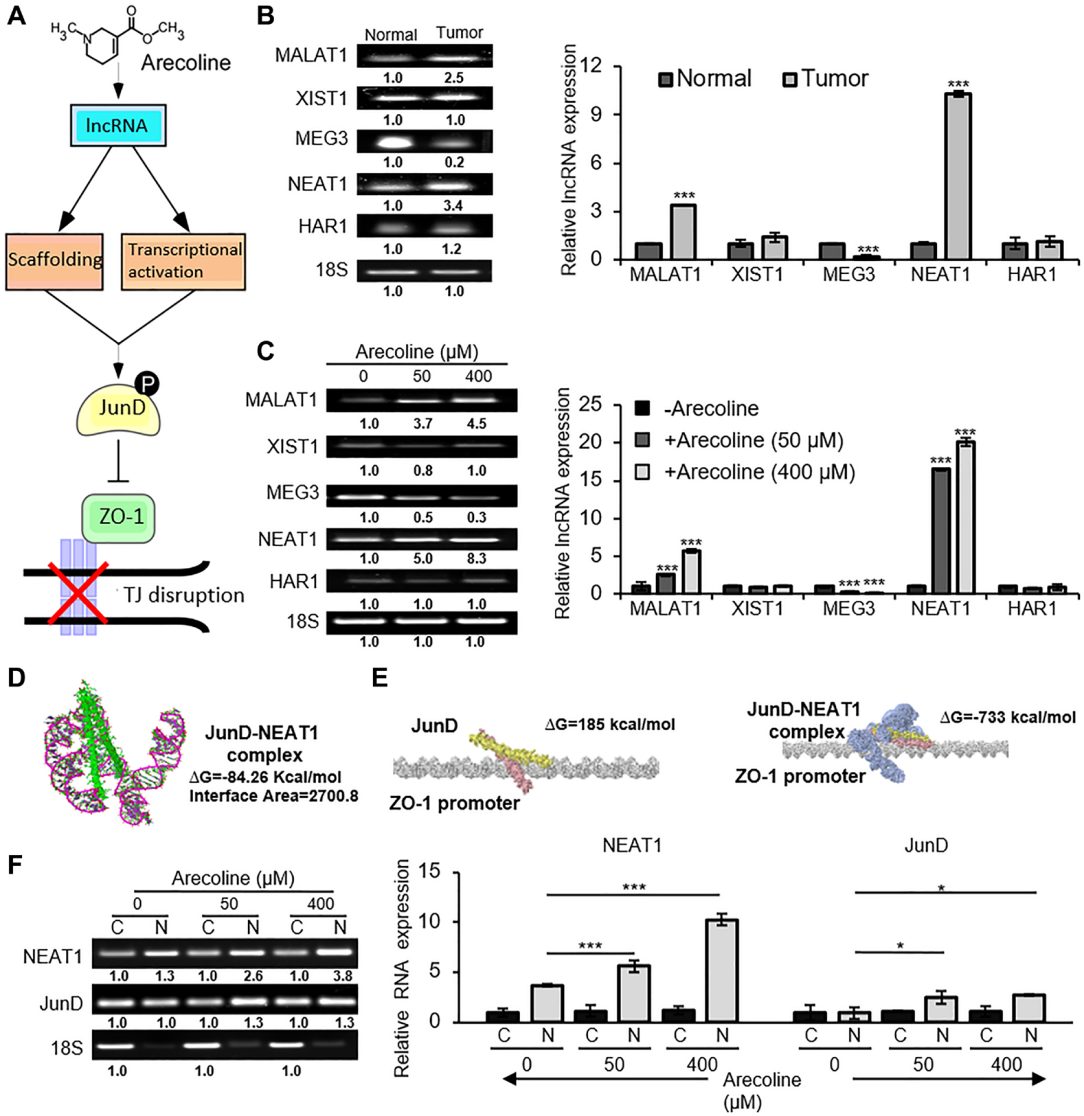
NEAT1 interacts directly with JunD in the nuclear compartment. (**A**) A putative schematic representation of arecoline-induced lncRNA-mediated activation of JunD and inhibition of ZO-1, leading to disruption of tight junctions. (**B**) Semi-qPCR (left) and qPCR assays (right) indicating differential expressions of lncRNAs in tumors and adjacent normal tissues of HNSCC patients. (**C**) Differential expressions of lncRNAs in HEp-2 cells treated without and with 50 and 400 μM concentrations of arecoline for 24 h. (**D**) Depiction of post-simulated NEAT1-JunD complex as evaluated from molecular docking using PatchDock software; NEAT1 lncRNA (pink ribbon model); JunD (green structure model). (**E**) Interaction of JunD and ZO-1 promoter (left) and NEAT1: pJunD: ZO-1 promoter (right) as evaluated from molecular docking studies using PatchDock. (**F**) Expression of NEAT1 and JunD mRNA in the cytosolic and nuclear fractions of HEp-2 cells treated without and with 50 and 400 μM arecoline. 18S rRNA expression was used as internal control for all PCRs. Each value is the mean ± S.D. of three different experiments. ^*^ *p* < 0.1, ^***^ *p* < 0.001.

Since JunD was differentially regulated in presence of arecoline we tried to figure out alternative mechanisms which regulated JunD activation. Subsequently, we investigated the involvement of lncRNAs as a plausible regulator. Long non-coding RNAs (lncRNAs) can interact with target proteins in different conformations and can act as scaffold for proteins to recruit them to their target region. They can also act to transcriptionally activate or deactivate a gene (Figure 7A). Several lncRNAs, known to be differentially regulated in HNSCCs as compared to normal oral squamous cells, were evaluated in normal versus tumor tissues (Figure 7B). Among the different lncRNAs, the nuclear paraspeckle assembly transcript 1 (NEAT1) was most significantly (*p* < 0.001) up regulated in human tumors. Treatment of HEp-2 cells with arecoline also revealed that expression of NEAT1 was dramatically increased at both the low and high concentrations (*p* < 0.001; Figure 7C). To explore the possible interaction of JunD with lncRNAs, RNA-Protein Interaction Prediction (http://pridb.gdcb.iastate.edu/RPISeq/) was employed to predict interaction probabilities. Subsequently, it was found NEAT1 and JunD in its phosphorylated form had the highest interaction probability of 95% using the SVM classifier. The computational docking studies of NEAT1 and JunD using PatchDock suggest that the binding has net negative energy of -84.26 kcal/Mol and the interface area of binding is 2700.80. Score of the model is 14492 and the rotational angles are -0.52 0.40 3.09 and the translational parameters are -2.96 -17.15 28.70 (Figure 7D). Further computational docking studies were performed to determine the interaction of JunD individually and in conjunction with NEAT1 to the ZO-1 promoter. Results indicated that NEAT1-JunD complex binding to the promoter region of ZO-1 has a net negative energy of -733kcal/Mol as opposed to 185 kcal/Mol for JunD alone, the interface area of binding being 3009.20 (Figure 7E). As a conformation to the interaction studies, sub-cellular fractionation assay demonstrated significant (*p* < 0.001) co-expression of NEAT1 and JunD in the nuclear fraction as compared to the cytosolic fraction, especially in the presence of arecoline (Figure 7F).

### Arecoline down regulates ZO-1 through NEAT1-JunD complex

**Figure 8:**
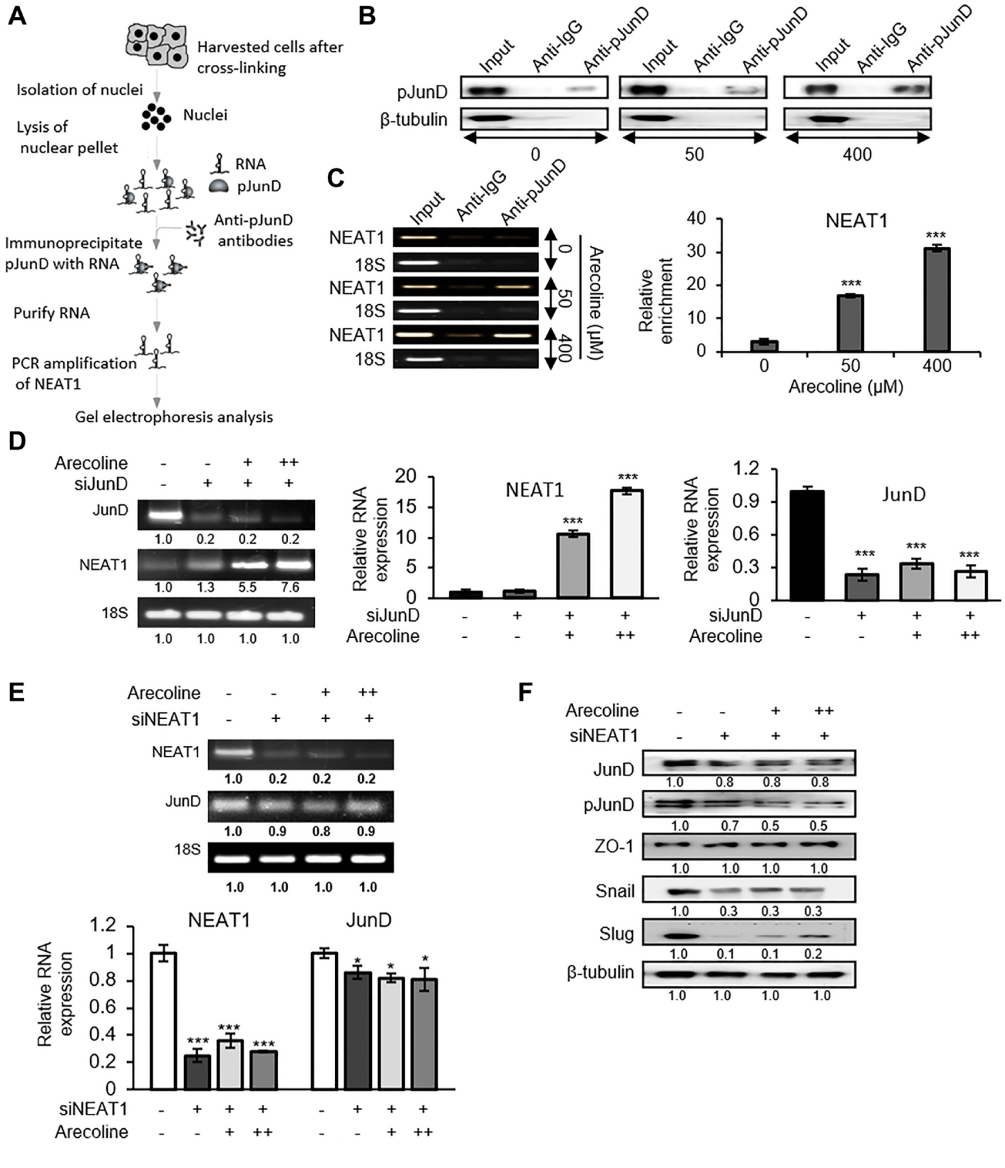
NEAT1 plays a pivotal role in JunD-mediated downregulation of ZO-1. (**A**) Schematic representation of RNA immunoprecipitation (RIP) performed to determine the interaction between pJunD and NEAT1. (**B**) Expression of pJunD in absence and presence of 50 μM and 400 μM arecoline after immunoprecipitation with pJunD specific antibody. RIP with anti-IgG, indicating non-specific antibody binding, served as the negative control. (**C**) Expression of NEAT1 following RIP by semi-qPCR and RT-PCR. 18S served as control for non-specific amplification. (**D**) Expression of JunD and NEAT1 after silencing JunD in HEp-2 cells followed by treatment without and with 50 μM (+) and 100 μM (++) arecoline. The relative RNA expressions are represented for NEAT1 and JunD. (**E**) Expression of JunD and NEAT1 after silencing NEAT1 in HEp-2 cells followed by treatment without and with 50 μM (+) and 100 μM (++) arecoline. (**F**) Differential protein expression of tight junction and EMT markers in HEp-2 cells after silencing NEAT1 followed by treatment without and with 50 μM (+) and 100 μM (++) arecoline. β-tubulin was used as an internal control for all western blots. All the experiments were performed three times. Each value is the mean ± S.D. of three different replicate experiments, each performed in triplicate. ^*^ *p* < 0.1 and ^***^ *p* < 0.001.

To further establish direct interaction between pJunD and NEAT1 in regulating ZO-1 expression, RNA-protein immunoprecipitation assay (RIP) was performed with the nuclear fraction of arecoline treated and untreated HEp-2 cells and pJunD specific antibody to precipitate NEAT1 lncRNA (Figure 8A). Figure 8B indicates that arecoline treatment enhanced pJunD, which precipitated with anti-pJunD antibody. That NEAT1 co-precipitated with pJunD was apparent when the above lysates were subjected to semi-q RT-PCR (Figure 8C). Although JunD silencing did not reduce NEAT1 expression in presence or absence of arecoline as shown by semi-quantitative RT-PCR and qPCR (Figure 8D), silencing NEAT1 significantly (*p* < 0.05) reduced the expression of JunD mRNA both in the presence and absence of arecoline (Figure 8E). Concomitantly, NEAT1 silencing specifically in the presence of arecoline, reduced pJunD significantly (*p* < 0.01), stabilized the expression of ZO-1 and significantly (*p* < 0.01) down regulated Snail and Slug (Figure 8F), emphasizing the essential role of NEAT1 in regulation of TJ proteins and EMT by arecoline.

## DISCUSSION

Betel nut chewing, one of the most popular addictive substances in the world which facilitates the digestive system and has mild euphoric effects, is consumed indiscriminately by men and women, children and adults [2]. Use of betel nuts is also associated with central obesity and type II diabetes. Further, the areca nut is carcinogenic in humans and is linked to cancers of the oral cavity and esophagus. Though the effects of areca nut are diverse, the molecular and cellular mechanisms of carcinogenicity of its major component, arecoline, have been moderately discerned till date [30]. Interestingly, the diverse activities of arecoline are tissue-specific and dose-dependent [31]. Our results indicated that arecoline, at lower concentrations, enhanced growth of HPV-positive HEp-2 cells whereas at higher concentrations was cytotoxic. This fact was further ratified in our experiments where we confirmed that a switch between the proliferative and apoptotic effects of arecoline lay somewhere between 90 and 110 μM when exposed for 24 hours, which included the concentration of arecoline in the saliva of betel nut chewers (23.7 to 415.2 μM), thereby defining both intermittent and regular usage as a risk for carcinogenic insult [8]. Significant results, however, were not observed with the HPV-negative SCC-131 cells. Previous studies have confirmed that comorbid conditions are known to have an additive impact on patients with OSCC [32] and that a high incidence of HPV infection-associated oral cancer has been seen in India among patients who chew betel quid [33]. Persistent HPV infection also plays an important role in enhancing the risk of betel nut chewing-associated cancers in Taiwan [6, 34], which may partially explain the insignificant effects of arecoline on SCC-131 cells compared to HEp-2 cells; hence, all mechanistic studies described here were carried out in the latter. Autophagy is known to facilitate tumorigenesis by promoting cancer cell proliferation and assisting cells to deal with stressful metabolic conditions [35]. In support, our results revealed increased formation of acidic vacuoles, as well as, enhanced expression of LC3 and Beclin1 in response to low concentrations of arecoline treatment. At higher concentrations (200–800 μM), however, arecoline induced G_2_/M cell cycle arrest at 24 and 48 h concomitant with enhanced cell death, possibly because of increased ROS generation simultaneously with deprivation of the antioxidant defense system [36].

Despite current advancement in cancer therapy, the 5-year overall survival rate of patients with HNSCC remains very low. The risk increases many folds for patients with nodal and distant metastasis as therapeutic options are limited [37, 38]. Metastasis entails cancer cells to overcome cellular barriers, intricately regulated by TJs, via programmed EMT facilitated by transcription factors like Slug, Snail and Twist along with over expression of N-cadherin [39]. Our results corroborate that expression of EMT-related factors are higher in tumors obtained from HNSCC patients compared to their normal counterparts, both at the transcriptional and translational levels, concomitant with down regulation of TJ-related genes and proteins. Similar outcomes were observed in HEp-2 cells treated with arecoline, in a dose- and time-dependent manner. Apparently controversial, since cell death too is enhanced at higher concentrations, the conundrum was resolved by an increase in the population of resistant stem-like cells (cancer stem cells), which are known to have increased EMT potentiality. Since arecoline activated several EMT-related factors, we analyzed if arecoline affected acquisition of stemness properties in cancer cells. Interestingly, several stemness-related markers were found to be up regulated in arecoline-treated cells, along with enrichment of ALDH^+^ population, indicating a greater likelihood of disease recurrence and metastasis if they were not obliterated during conventional therapy.

To ascertain the molecular pathway regulating arecoline-induced TJ disassembly, we screened for putative regulators involved in the TJ signaling pathways by differential gene expression. Data mining indicated that JunD, a transcription factor binding AP-1 sites and implicated in various tumor types [12], may be responsible for regulating ZO-1, which is indispensable for TJ assembly and function [40]. Subsequently, both tumor tissues of HNSCC patients and arecoline-treated cells showed over expression of and activation of JunD by phosphorylation, along with down regulation of ZO-1. Further, silencing JunD restored the expression of ZO-1, confirming an antagonistic role of JunD on ZO-1. JunD activation therefore enhanced the metastatic potential of cancer cells, since in the presence of JunD, arecoline sequestered ZO-1 from the membrane to the cytosolic fraction of the cells, thereby lifting the cell barrier.

Since phosphorylation is reportedly a major mechanism regulating TJ integrity [15], we investigated whether phosphorylation of JunD is the lone event which can regulate ZO-1 in response to arecoline. Based on a single report describing the role of JunD in regulating intestinal epithelial barrier function [41] and since pJunD was elevated in tumor samples, we checked the status of JunD phosphorylation in HEp-2 cells in addition to various components of the MAP kinase signaling pathway, such as PI3K, JNK and AKT. Subsequently, we observed arecoline-induced elevated levels of pJunD, along with pJNK and pAKT, in HEp-2 cells in a dose-specific manner. In order to determine whether PI3K, AKT and JNK served as components upstream of JunD which regulated ZO-1 expression, HEp-2 cells were treated with and without wortmannin, a known inhibitor of PI3K, in the absence and presence of arecoline. As expected, inhibiting PI3K prevented phosphorylation of AKT, JNK and JunD in the absence of arecoline. However, in presence of arecoline, even though phosphorylation of AKT and JNK was inhibited, JunD phosphorylation was enhanced, together with reduced expression of ZO-1. Further, silencing JunD did not affect expression of the upstream effectors. This observation led to the understanding that, in the presence of arecoline, phosphorylation by upstream components is not the sole event for activation of JunD and prompted investigation for alternate mechanisms.

LncRNAs are known to modulate gene expression and exert cellular effects through diverse mechanisms. They can interact with DNA, RNA and/or proteins in multiple configurations depending on the secondary and tertiary structures [42]. Screening for differential lncRNA expression pattern in tissues of HNSCC patients revealed that NEAT1, MALAT1 and MEG3 are highly deregulated in the tumors compared to normal tissues. Of the three lncRNAs, NEAT1 (Nuclear Paraspeckle Assembly Transcript 1), which is known for its oncogenic role in many cancers including LSCC (lung squamous cell carcinoma) and HNSCC, was highly up regulated in response to arecoline treatment. Interestingly, inhibiting NEAT1 significantly inhibited tumor growth in patients with neck nodal metastasis [43], emphasizing its value as a therapeutic target. Although our results indicated that different concentrations of arecoline demonstrated opposing effects of cell proliferation versus cell death, many of the biochemical changes involving the lncRNAs were found to be similar at both low and high concentrations of arecoline, albeit with differential intensities. This may be possible since NEAT1 has been shown to be involved in diverse biochemical events, such as EMT, autophagy and proliferation, in various cell models [44, 45]. In accordance, it was imperative to evaluate whether NEAT1 coordinated with JunD in response to arecoline and modulated ZO-1 expression. Interaction prediction and modeling software indicated that binding of NEAT1 and JunD was highly probable and energetically favorable. Interaction analyses also indicated that binding of NEAT1-JunD complex to the ZO-1 promoter is energetically far more favorable than binding of JunD alone. Since binding of JunD to the ZO-1 promoter has been previously demonstrated [41], it may be conjectured that this binding is in all probability enhanced in the presence of NEAT1 lncRNA, since individually NEAT1 and JunD are enriched in the nuclear fraction in response to arecoline treatment. However, since individual over expression does not ensure interaction, an RNA immunoprecipitation assay was performed to assess definitive interaction between the two. The results substantiated specific association of NEAT1 lncRNA with JunD in the nuclear fraction in the presence of arecoline, confirming the alternative mechanism by which arecoline modulated JunD in HEp-2 cells. Although silencing JunD did not reduce the expression of NEAT1 in presence or absence of arecoline, silencing NEAT1 suppressed the expression of JunD, emphasizing the importance of NEAT1 association with JunD to render it functional and bind to the ZO-1 promoter. In addition, silencing NEAT1 in the presence of arecoline not only stabilized ZO-1 expression, it reduced the expression of EMT markers, indicating possible re-establishment of TJs and prevention of metastasis. Therefore, in cancer cells exposed to arecoline, JunD is activated not by phosphorylation alone but by interaction with NEAT1 to suppress the expression of ZO-1 and destabilize structural integrity of TJs.

## MATERIALS AND METHODS

### Reagents

Microarray kit (PAHS_143Z), RT^2^ first strand kit and RNA later™ were purchased from Qiagen (USA). Minimum Essential Medium (MEM), fetal bovine serum, 0.25% trypsin-EDTA, and 100X antibiotic and antimycotic mix were from HiMedia (India). Arecoline hydrobromide, 3-(4,5-dimethylthiazol-2-yl)-2,5-diphenyl tetrazolium bromide (MTT) kit, 2′,7′ –dichlorofluorescin diacetate (CM-H_2_DCFDA), sodium fluoride (NaF) and N-acetyl-L-cysteine (NAC) were purchased from Sigma-Aldrich (USA). Antibodies were purchased from Santa Cruz (USA) and Abcam (USA). The list of all the antibodies and primers used are provided in Supplementary Tables 1 and 2, respectively. TRIzol^®^, reverse transcription kit Superscript-RT and lipofectamine was purchased from Invitrogen (USA). KAPA SYBR FAST qPCR KIT Master Mix (2X) was procured from Kapa Biosystems (USA).

### Histological and immunofluorescence analysis of tissues

HNSCC tumor and their paired histopathologically normal tissue samples (*n* = 56) were collected from patients at Silchar Medical College, Assam, India, as per the directives of the Institutional Review Board and in accordance to the guidelines of the Institutional Human Ethical Committee. Informed consent was collected from patients and a history of betel nut use was recorded. The tumors (mostly from stages T3 and T4) were exclusively primary-site cancers, from different sites of head and neck cancers (buccal mucosa, tongue, hypopharynx and larynx), that were either naïve or had been subjected to chemotherapy prior to surgery. Normal tissues were collected 6 cm away from or a site opposite to the tumor site. Integrity of the tissues was confirmed by trained pathologists.

After washing the tissues in ice-cold PBS, tissues were preserved in RNA later™. Portions of normal oral and oral tumor tissues were removed aseptically and fixed in 10% buffered formalin for 24 h, dehydrated and then embedded in paraffin. 5 μ sections were stained with hematoxylin and eosin. For immunofluorescence staining, sections were incubated overnight at 4°C with primary antibody diluted in PBS (1:100) followed by incubation in secondary antibodies (1:100) at room temperature for 2 h. Sections were mounted in Gel Mount and viewed under a FV 1200 confocal laser scanning microscope (Olympus, USA) [46].

### Cell culture

Human epithelial-type laryngeal carcinoma cell line, HEp-2, was procured from National Centre for Cell Science (NCCS), Pune, India and routinely maintained in high glucose MEM media. SCC-131 oral squamous carcinoma cells were maintained in DMEM:F12 (1:1) media supplemented with 10% FBS and 1X antibiotic and antimycotic mix in 95% humidified air, 5% CO_2_ at 37°C. At 90% confluency, the cells were dissociated with 0.25% (w/v) trypsin/EDTA and sub-cultured. Cells were routinely tested by PCR for presence of any contamination [47, 48]. Before each passage, cell viability was checked with trypan blue dye exclusion test and cells in the log phase were used for subsequent experiments.

### Drug treatment

Arecoline was dissolved in DMSO and kept as a 1M stock solution. HEp-2 cells were treated with various concentrations of arecoline ranging from 5 μM to 1000 μM for specific periods of time. The control set was treated with an equal volume of DMSO. The concentration of DMSO was kept below the permissible limit of 1% in all the experimental sets. The concentrations of arecoline used conformed to that which generally ranged in the saliva (23.7 to 415.2 μM) of regular betel nut chewers (8) and were also confirmed to achieve an IC_50_ through preliminary dose-response experiments using cell lines.

### Reverse Transcription (RT)-PCR and Real-Time PCR analysis

Cells treated with arecoline for specific periods of time and biopsy samples from patients (*n* = 5) were used for RT-PCR analysis. Total RNA was purified from samples using TRIzol^®^ according to the manufacturer’s instructions. For mRNA analysis, complementary DNA of each sample was randomly primed from 1 μg of total RNA using Superscript-RT. Real time PCR was performed using the KAPA SYBRFAST qPCR KIT. Data was normalized to18s rRNA and relative expression levels were determined using StepOne Real Time PCR software. Semi-quantitative PCR analysis was carried out in a total volume of 10 μl containing 0.5 picomoles of each primer using Go Taq Flexi DNA Polymerase on the 2720 Thermal Cycler (Applied Biosystems, USA) The relative quantification value for each target gene was expressed as 2^−ΔΔCT^ [49].

### Subcellular fractionation

The treated and untreated cells were lysed with a subcellular fractionation (SF) buffer. The pellet (nuclear fraction) was washed and resuspended in nuclear (NL) buffer. The supernatant was centrifuged at 10,000× g at 4°C for 10 min, followed by ultra-centrifugation at 100,000× *g* at 4°C for 1 h. The supernatant was collected as the cytoplasmic fraction. The pellet (microsomal fraction) was washed with SF buffer and resuspended in NL buffer [50].

### Western blot analysis

Arecoline treated and untreated cells and tissue samples were harvested in RIPA buffer containing protease inhibitor cocktail (Abcam, USA). Equal amounts of total cellular, cytosolic and microsomal proteins were fractionated by 10% polyacrylamide gel electrophoresis and transferred on to PVDF membranes. Protein blots were subsequently incubated overnight at 4°C with primary antibodies. Blots were subsequently incubated with HRP-tagged secondary antibodies and bands detected using chemiluminescence in the Gel Doc XR type imaging system (BioRad, USA). The results were quantified using ImageJ software (https://imagej.nih.gov/ij/) and expressed as fold change relative to the control after normalization with β-tubulin [49].

### Cell viability assay

Cells were plated at a density of 1 × 10^4^ cells per well in a 96-well plate and exposed to medium containing different concentrations of arecoline (0 to 1000 μM). After incubation, a final concentration of 0.5 mg/ml MTT was added to each well. Formazan produced by viable cells was dissolved in DMSO and measured at 570 nm against blank using Multiskan™ GO microplate spectrophotometer (Thermo Scientific, USA) [49].

### Cell cycle analysis

Cells were treated without and with arecoline, trypsinized and washed with cold PBS. Cells were subsequently fixed, treated with 20 μg/ml RNase and stained with 50 μg/ml propidium iodide in PBS for 30 min on ice. Distribution of cells in different phases of cell cycle was characterized by flow cytometric analysis using an Accuri C6 flow cytometer (BD Biosciences, USA) and cell cycle profiles were analyzed by the BD Accuri C6 software (BD Biosciences, USA) [49].

### 4′,6-Diamidino-2-phenylindole (DAPI) staining

Following treatment with arecoline, cells were fixed in 80% ethanol for 30 min at room temperature and stained with DAPI (0.5 μg/ml in PBS) for 1 min. Cells were observed and documented using a EVOS FL fluorescent microscope (Thermo scientific, USA) [51].

### Aldefluor assay for stem cell detection

Aldehyde dehydrogenase (ALDH) activity in viable and intact stem cell population was determined using the activated fluorogenic dye based aldefluor assay. 1 × 10^6^ cells were resuspended in assay buffer containing the ALDH substrate and incubated for 45 min at 37°C. The reference samples were suspended in buffer containing diethylaminobenzaldehyde (DEAB), a specific ALDH1 enzyme inhibitor in addition to the aldefuor substrate. The ALDH^high^ population was detected in the green fluorescence channel (520–540 nm) of FACS Aria III (BD Biosciences) and sorted [49].

### Spheroid formation assay

Sphere-forming capacity of the cancer stem cells was evaluated by plating 1 × 10^4^ cells per well of 6-well ultralow attachment plates in serum-free MEM containing 5 μg/mL bovine insulin, 20 ng/mL recombinant epidermal growth factor, B27 supplement, and antibiotic-antimycotic mix. The plates were incubated at 37°C for 3 days and formation of spheroids was confirmed by observing under the microscope [49].

### Immunocytochemistry

Cells were grown on glass cover slips and were exposed to arecoline for 24 h. Samples were fixed in 3.7% paraformaldehyde in PBS for 15 min at room temperature, permeabilized with 0.1% Triton X-100 in PBS for 15 min, and blocked with 5% horse serum in PBS for 2 h. Cells were incubated with primary antibody in PBS (1:100, 1 h) in a moist chamber, followed by incubation in FITC-tagged secondary antibody for 1 h and DAPI for 30 sec. Cover slips were fixed with Gel Mount and visualized under a FV 1200 confocal laser scanning microscope (Olympus, USA) [49].

### Trans-epithelial electrical resistance (TEER) measurement

HEp-2 cells was plated in 12-mm, 3-μm-pore-size polycarbonate filter inserts at a density of 10^5^ cells/insert (Millipore, MA). Following a 3-day incubation to allow the cells to become confluent and form tight junctions, cells were exposed to varying concentrations of arecoline and incubated at 37°C in a 5% CO_2_ environment for 24 and 48 h. TEER measurements were performed immediately prior to and following the addition of arecoline at respective time points using an EVOM Epithelial Volt-ohmmeter (World Precision Instruments) to ensure polarization of the monolayer. The TEER values (Ω x cm^2^), done in triplicates and repeated twice, were calculated by subtracting the mean resistance of control inserts (blank) from the mean resistance of cells treated with various concentrations of arecoline at given time points and normalized to the growth area of the monolayer [52].

### Wound healing assay

Cells were seeded in 6-well plates at 10^5^ cells/ml. When the cells reached 80–90% confluency, the monolayer was scraped in a straight line to create a ‘scratch’. Cells were washed twice with PBS and then replaced with media containing arecoline for the treatment set and regular media for the control set. Migration of cells was recorded after 24 and 48 h of treatment [49].

### Proliferation analysis

Cells were harvested after treatment with arecoline and fixed in 80% ethanol at –20°C for 2 h. Cells were washed twice and resuspended in staining buffer (PBS with 1% FBS, 0.09% NaN_3_). Approximately 10^6^ cells were stained with Ki-67 antibody (1:100) in the dark at room temperature for 30 min. Cells were finally washed and resuspended in PBS prior to flow cytometric analysis [53].

### Autophagy detection assay

Arecoline treated and untreated cells were harvested and resuspended in PBS. The cells were then incubated with acridine orange (1 μg/ml) for 15 min, washed and re-suspended in fresh PBS. The green/red fluorescence was detected using BD FACS Verse and autophagy positive cells, as indicated by formation of acidic vesicular organelles (AVO), were quantified using CellQuest^®^ software (BD Biosciences, San Jose, USA) [46].

### Apoptosis detection assay

Cells were harvested and resuspended in 1X binding buffer (Abcam, USA). They were incubated with Annexin V–FITC and PI staining solution for 15 minutes at 37^°^C in the dark and analyzed using a flow cytometer (BD Biosciences, USA). The percentages of apoptotic and necrotic cells were calculated using BD ACCURI C6 software [46].

### Measurement of ROS levels

Arecoline treated and untreated cells were incubated with 10 μM CM-H_2_DCFDA. After 15 minutes, cells were washed and resuspended in PBS. The levels of fluorescence were detected immediately using flow cytometry. For fluorescence imaging, cells were seeded in a 6-well plate and treated with different concentrations of arecoline for 24 hrs. Cells were washed, fixed in 4% paraformaldehyde and incubated with 5 μM CM-H_2_DCFDA for 30 minutes. Images were recorded in an EVOS FL fluorescent microscope (Thermo scientific, USA) [54].

### RT² profiler PCR array

Samples were processed for total RNA extraction and 1 μg of total RNA was converted to cDNA. Samples were prepared for RT² Profiler PCR Array for assessing expression of 84 key genes, responsible for encoding proteins associated with Human Tight Junctions. Expression profile of the tumor tissue was compared to non-tumor tissue, procured 6 inches away from the site of the tumor of the same patient and confirmed by a pathologist. Microarray data was analyzed with Gene Globe Data Analysis Center (Qiagen, USA). The data were normalized against housekeeping genes and used to determine the fold change. Genes which were differentially expressed between the two groups were identified and selected genes were further categorized according to their gene ontology annotations [49].

### siRNA transfection

HEp-2 cells were seeded in 6-well plates 24 h prior to transfection at a cell density of 10^5^ cells. For silencing JunD, siRNA duplex was added. Unspecific scrambled siRNA duplex was used as the negative control. The transfection of the cells was performed using lipofectamine according to manufacturer’s instructions [49].

### Molecular modeling

The protein binding region of NEAT1 (1001–1540) was chosen according to Wang et al. [55] and the 3D molecular structure of the core region was predicted using SimRNAweb. The model with an optimal binding geometry was chosen. The structure of JunD was obtained from SwissProtPDB. The 3D structure of the ZO-1 promoter region was predicted using SCFbio webserver. PatchDock was used for analysis of the interaction of JunD with NEAT1 in order to resolve whether JunD alone or JunD in complex with NEAT1 interacts with the promoter region of ZO-1. PatchDock program divides the interacting molecules into segments and search for shape complementarities in the resulting surfaces. The quality of the fit is assessed and scored and the binding energy is calculated based on the desolvation of the interfaces. The top 20 solutions from each round of calculations were manually checked for interaction sites and orientation of the molecules and the most energetically stable conformation was chosen for further studies.

### Cycloheximide assay

The cells were seeded at a density of 10^5^ cells per well in a 6-well plate. For determination of the stability of JunD in presence of arecoline, cycloheximide was added to the cultures at a final concentration of 100 μg/mL, both in the presence and absence of arecoline. After 6 hrs, the reaction was stopped and cells were collected for analysis by western blot analysis [56].

### RNA immunoprecipitation-qPCR assays

To determine whether NEAT1 and JunD interact directly, bioinformatic analysis was performed using RNA-Protein Interaction Prediction (RPISeq). The prediction was based on the RF (random forest) and SVM (support vector machine) classifiers. Based on the prediction RNA immunoprecipitation (RIP) was performed according to manufacturer’s instructions. Anti-JunD antibody was used for the experiments. The lysates were incubated with the antibody overnight at 4^°^C and the co-precipitated RNAs were detected by qPCR [44].

### Statistical analyses

All the data in this study was analyzed by Graph Pad Prism 5.0 (GraphPad Software Inc., USA) and expressed as mean ± SD. Comparison between two groups was done by the paired Students’ *t*-test, and one-way ANOVA followed by Tukey’s post hoc test for multiple group comparisons. Differences was considered significant at *p* < 0.05. Densitometric analyses of western blot and cell population after FACS analyses were represented by bar diagrams. A minimum of three independent experiments for each protocol was conducted to allow for valid statistical comparisons [49].

## CONCLUSIONS

In summary, lower concentrations of arecoline promoted proliferation, induced EMT, augmented stemness acquisition and ensured cancer cell survival through autophagy. At higher concentrations, arecoline led to cell cycle arrest and apoptosis of the cancer cells but sustained the cancer stem cell population and enhanced EMT markers, supporting metastasis and possibility of disease recurrence, thereby reiterating the enhanced risk of tumorigenesis in both occasional and chronic betel nut chewers with lesions or comorbidities. Further, both low and high concentrations of arecoline endorsed favorable coupling of JunD and NEAT1 to repress expression and recruitment of ZO-1 at the cell membrane, leading to disruption of tight junctions. Thereby, strategies to stabilize tight junctions and prevent metastasis need to address designing molecules which would target NEAT1 in order to prevent activation of JunD by NEAT1 lncRNA.

## SUPPLEMENTARY MATERIALS



## Abbreviations

TJ: tight junctions
EMT: epithelial-mesenchymal transition
ZO-1: Zonula occludens 1
NEAT1: Nuclear Enriched Abundant Transcript 1
FACS: Fluorescence-activated cell sorting
FITC: Fluorescein isothiocyanate
MTT: 3-[4,5-dimethylthiazole-2-yl]-2,5-diphenyltetrazolium bromide
DMSO: Dimethyl sulfoxide
PVDF: Polyvinylidene difluoride
