# Supplementary material for: JunD accentuates arecoline-induced disruption of tight junctions and promotes epithelial-to-mesenchymal transition by association with NEAT1 lncRNA
Source: Oncotarget. 2021 Jul 20;12(15):1520–39. doi: 10.18632/oncotarget.28026 (PMC8310672; doi:10.18632/oncotarget.28026)
Supplement: Supplementary file 1 [file oncotarget-12-1520-s001.pdf]

JunD accentuates arecoline-induced disruption of tight junctions and promotes epithelial-to-mesenchymal transition by association with NEAT1 lncRNA

SUPPLEMENTARY MATERIALS

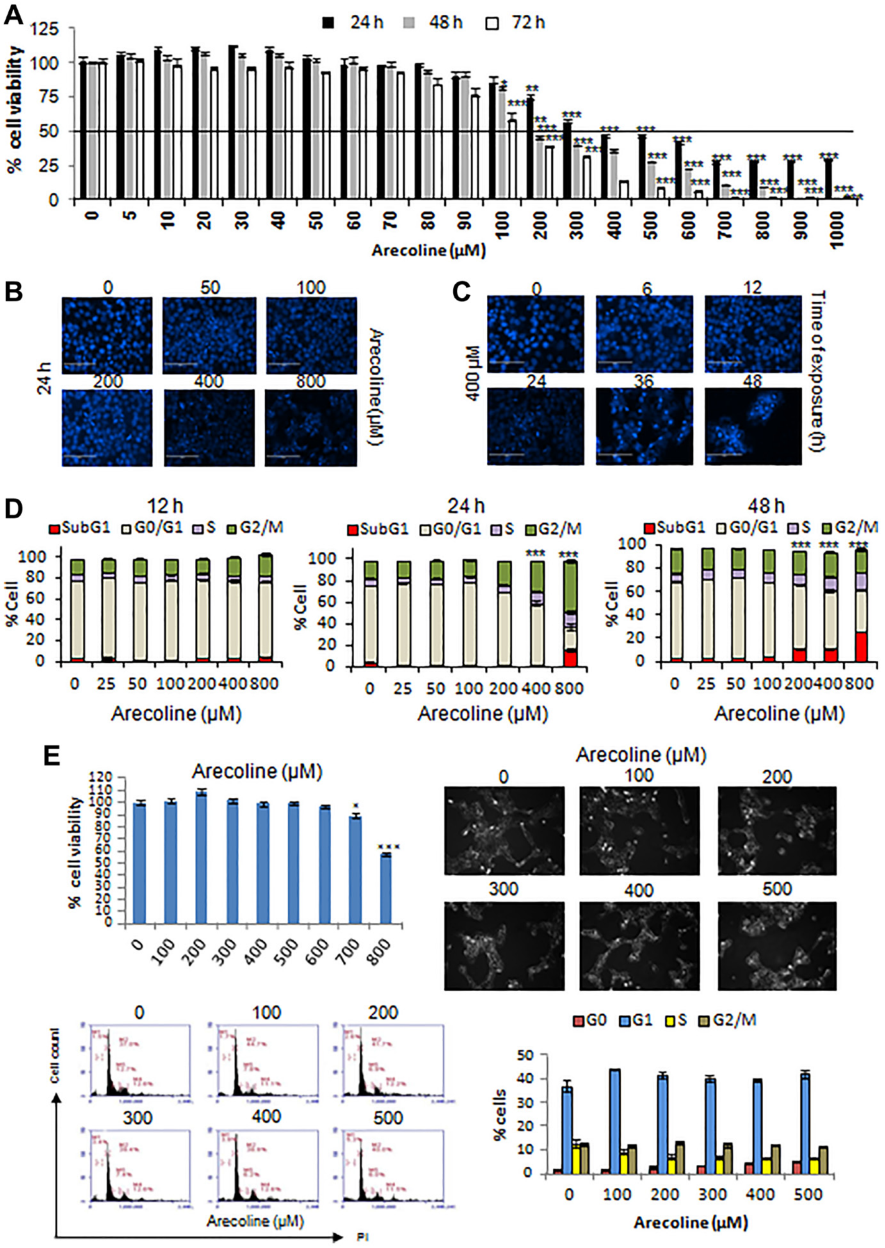

Supplementary Figure 1: (**A**) Effect of arecoline on cell viability of HEp-2 after 24, 48 and 72 h of exposure as determined by MTT assay. (**B**) The fluorescent micrographs of nuclear-stained HEp-2 cells are depicting the dose-dependent effect of arecoline treatment on the morphology of the nucleus. The nuclei were stained with DAPI. Scale bar: 100 µm. (**C**) Fluorescent micrographs of HEp-2 cells depicting the time-dependent effects of arecoline treatment on the morphology of the nucleus. Scale bar: 100 µm. (**D**) Quantitative representation of phases of cell cycle in HEp-2 after 12 h, 24 h and 48 h of arecoline treatment. (**E**) Effect of various doses of arecoline on cell viability (upper left panel), cell proliferation (upper right panel) and cell cycle (lower panel) of SCC-131 cells after 24 h of exposure as determined by MTT assay, phase contrast image of cells and cell cycle analysis, respectively. All the experiments were performed three times. Each value is the mean ± S.D. of three different replicate experiments, each performed in triplicate. $^{*}p < 0.1$, $^{**}p < 0.01$, and $^{***}p < 0.001$.

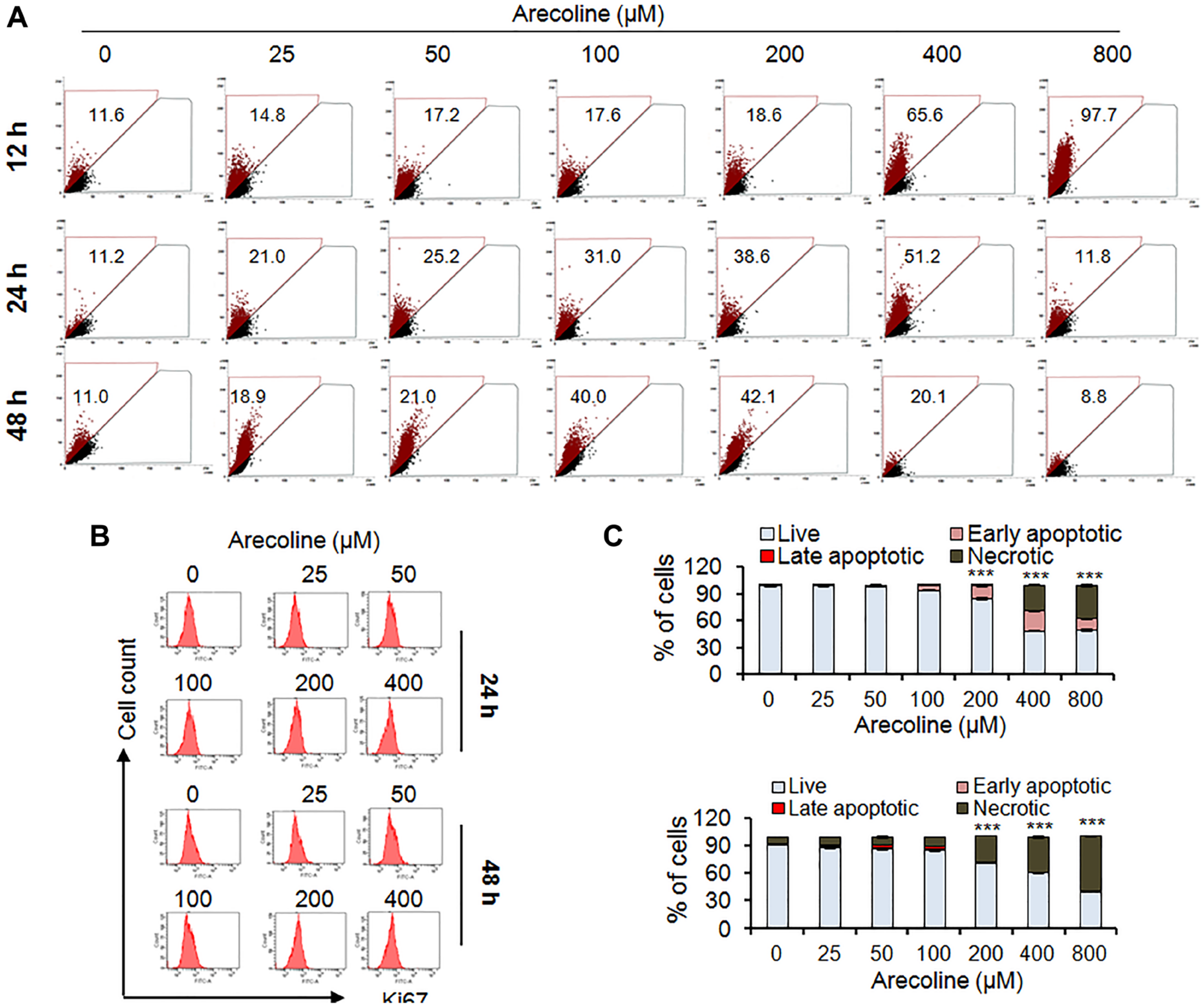

Supplementary Figure 2: (**A**) Effect of various concentrations of arecoline on induction of autophagy in HEp-2 cells after 12, 24 and 48 h of exposure, as indicated by increased formation of AVOs. The fluorescent intensities indicated by PI fluorescence (x-axis) versus the number of cells (y-axis). (**B**) Effect of arecoline treatment for 24 h (upper panel) and 48 h (lower panel) on HEp-2 cell proliferation as indicated by increased Ki-67. The fluorescent intensity of FITC was determined by flow cytometry and plotted in the semi-logarithmic graph of FITC fluorescence (x-axis) versus the number of cells (y-axis) (**C**) Quantitative representation of apoptosis assay in HEp-2 cells after 24 h (top panel) and 48 h (bottom panel) of arecoline treatment. Each value is the mean ± S.D. of three different replicate experiments, each performed in triplicate. $^{*}p < 0.1$, $^{**}p < 0.01$, and $^{***}p < 0.001$.

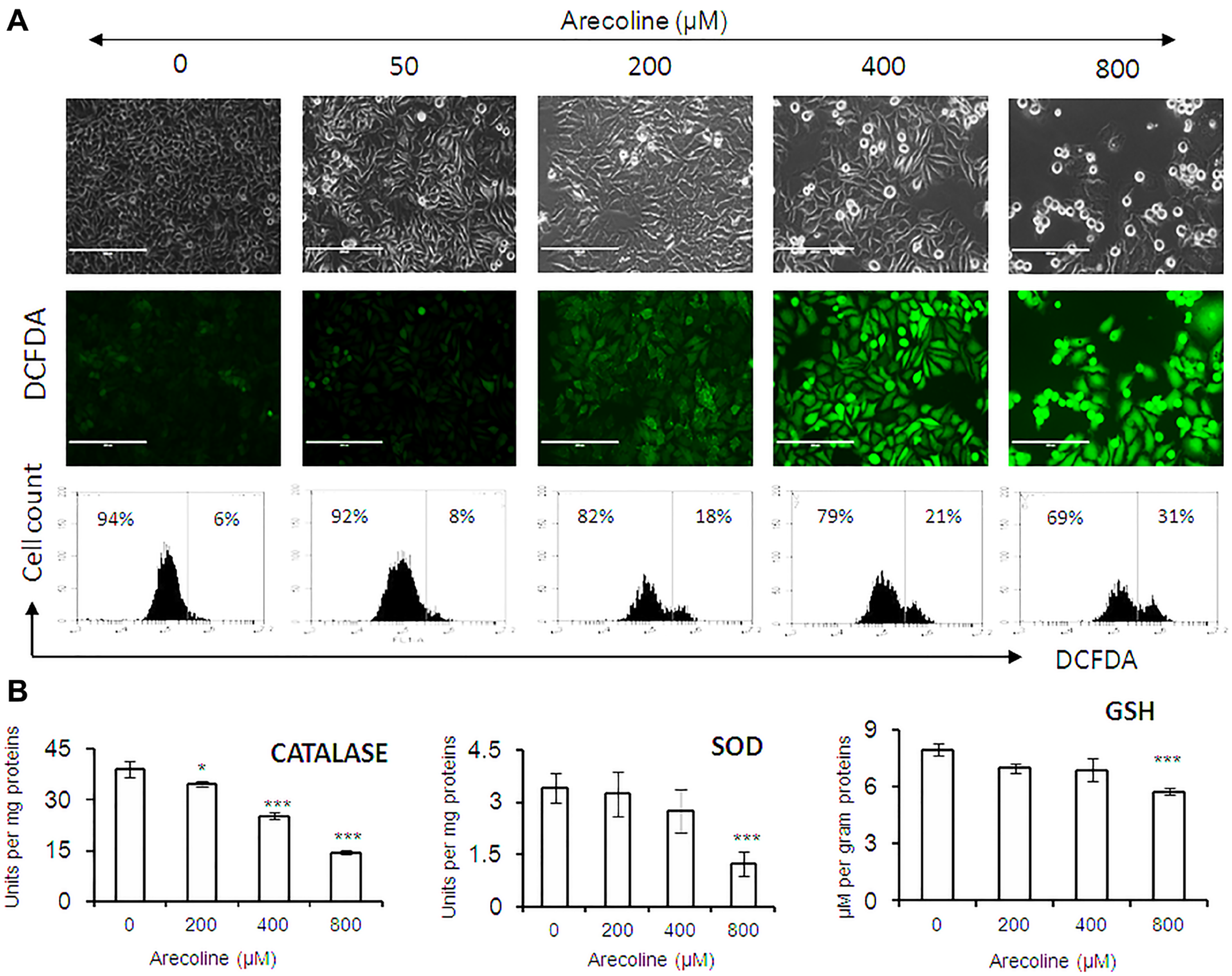
A
Arecoline (μM)
0
50
200
400
800
DCFDA
Cell count
94%
6%
92%
8%
82%
18%
79%
21%
69%
31%
DCFDA
B
CATALASE
Units per mg proteins
45
30
15
0
200
400
800
Arecoline (μM)
SOD
Units per mg proteins
4.5
3
1.5
GSH
μM per gram proteins
9
6
*
***

C

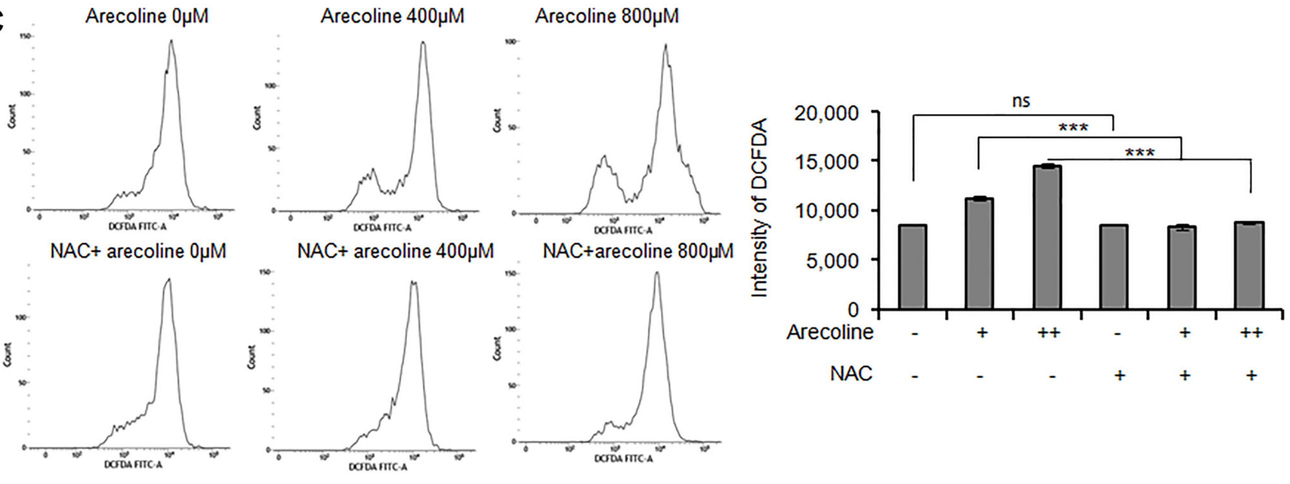

D

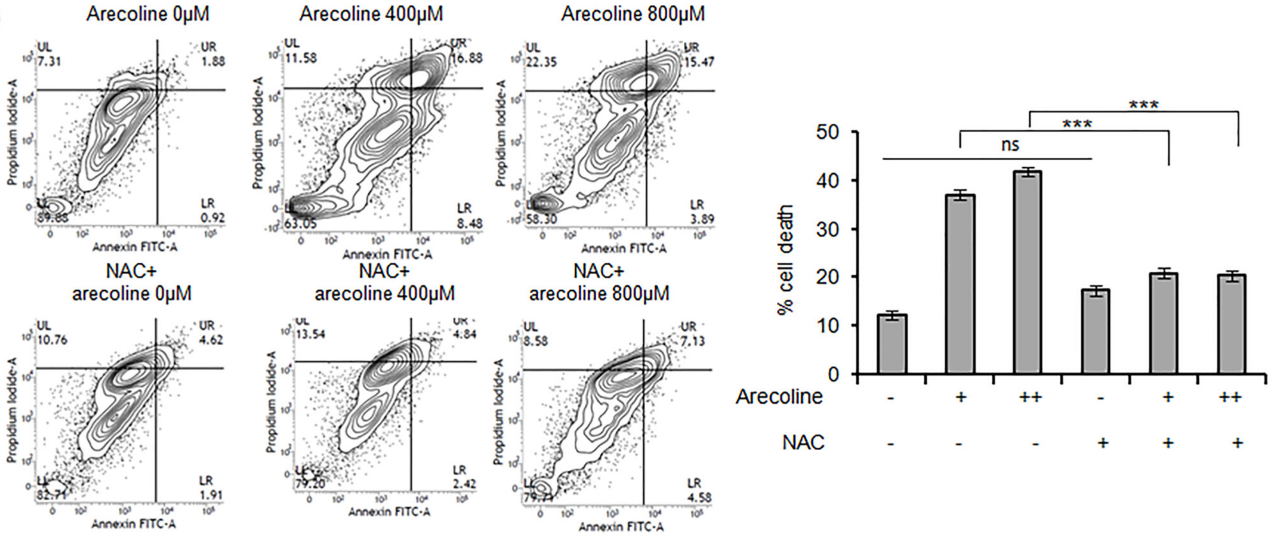

Supplementary Figure 3: (**A**) ROS generation in Hep-2 cells in response to different doses of arecoline treatment for 24 h, as evaluated by fluorescence imaging (middle panel) and flow cytometry (lower panel). The upper panel shows the phase-contrast micrographs of arecoline treated Hep-2 cells. (**B**) Activities of antioxidant defence components, catalase, superoxide dismutase (SOD) and glutathione (GSH) in response to treatment with different doses (0, 200, 400 and 800 μM) of arecoline for 24 h. (**C**) ROS generation in response to the absence and presence of 20 mM NAC for 24 h, without and with 400 μM (+) and 800 μM (++) arecoline. The graphical representation of the same is shown alongside. (**D**) Apoptosis assay indicating death of HEp-2 cells in response to different doses (0, 400 and 800 μM) of arecoline for 24 h in the absence and presence of 20 mM NAC. The graphical representation of the same is shown alongside.

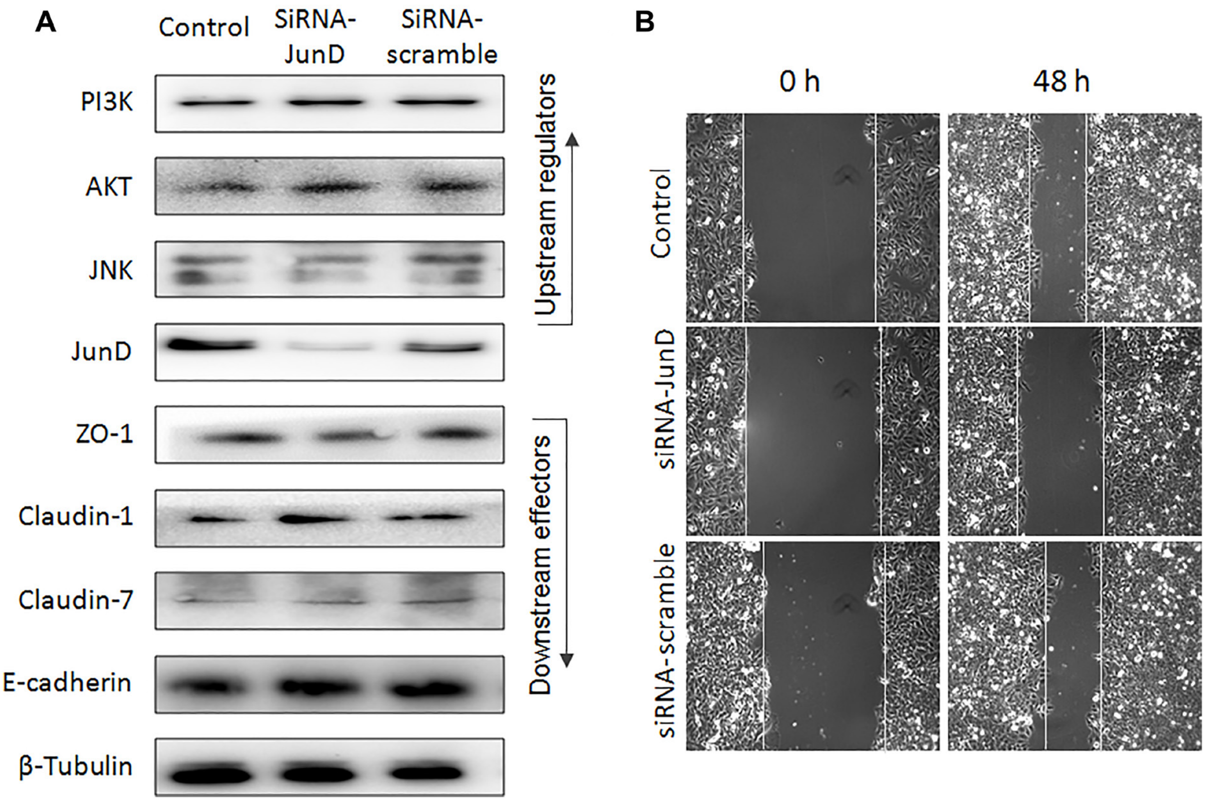

Supplementary Figure 4: (**A**) Effects of JunD-siRNA and scramble siRNA on tight junction and MAPK-related proteins in HEp-2 cells. β-tubulin was used as an internal loading control. (**B**) Bidirectional wound healing assay illustrating the effects of JunD silencing on HEp-2 cells in contrast to scramble siRNA after 48 h of incubation.

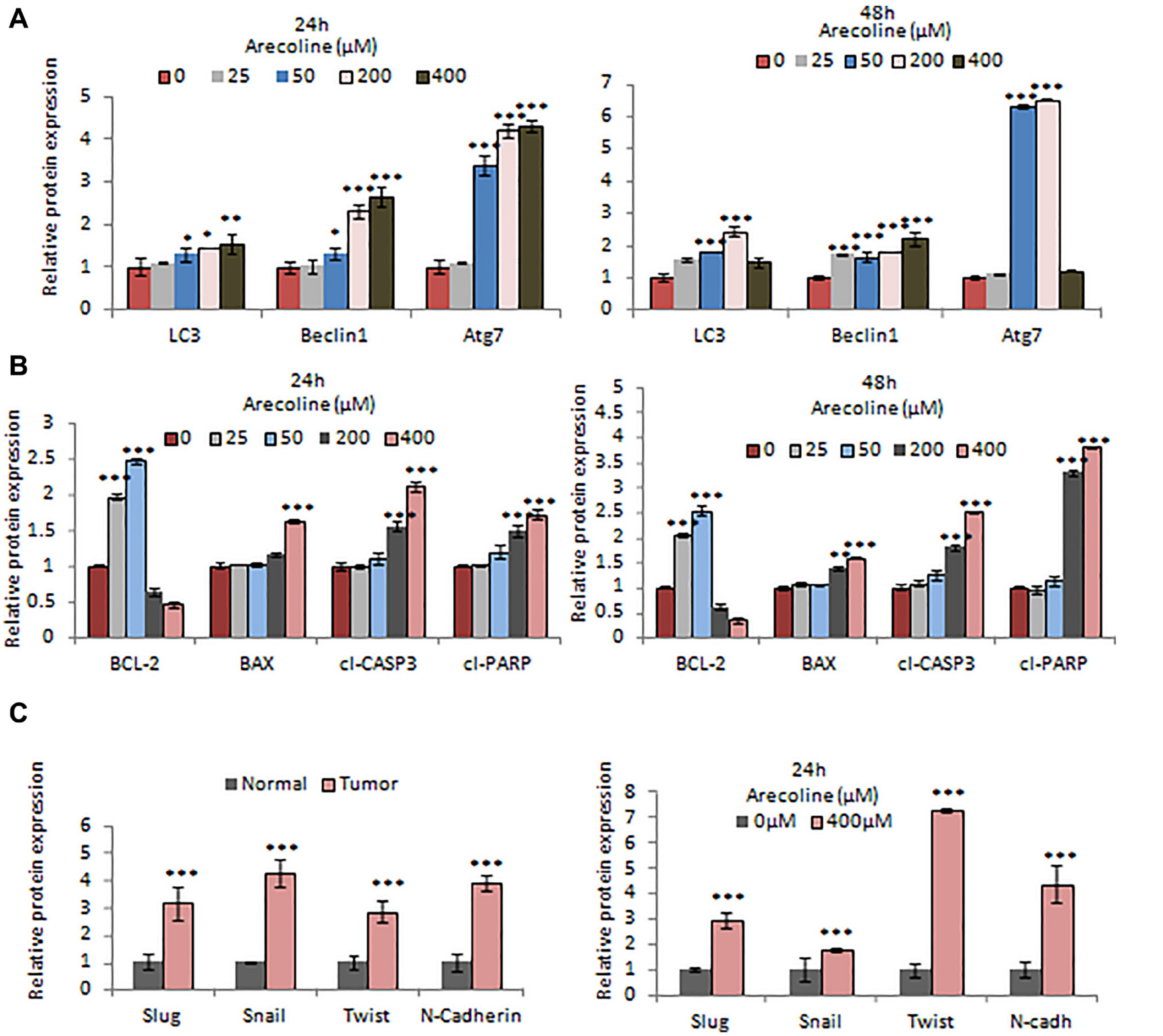

Supplementary Figure 5: (**A**) Quantification histograms showing increased expression of autophagy-related proteins LC3-II, Beclin1 and Atg7 in HEp-2 cells upon treatment with arecoline in dose-dependent manner (0, 25, 50, 200, 400 µM) for 24 h (left panel) and 48 h (right panel). (**B**) Graphical representation of apoptosis-related proteins such as Bcl-2, Bax, cleaved caspase 3 (cl-caspase 3) and cleaved PARP (cl-PARP) in HEp-2 cells upon treatment with different concentrations of arecoline for 24 h (left panel) and 48 h (right panel). (**C**) Quantitative analysis showing expressions of Snail, Slug, Twist and N-cadherin proteins in oral tumor tissues and adjacent normal tissues of HNSCC cancer patients (left panel) and in arecoline (0, 400 µM) treated HEp-2 cells (right panel).

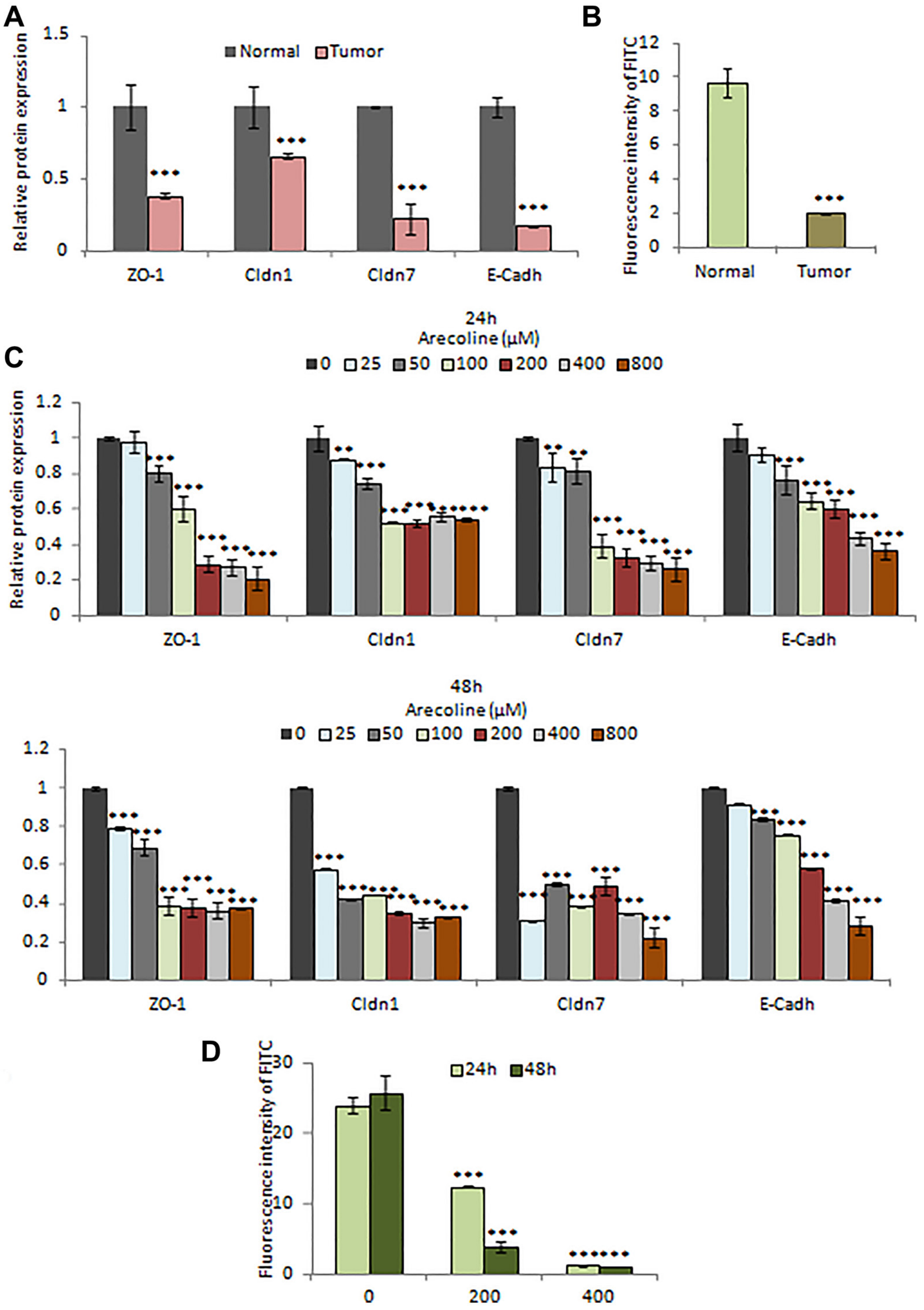

Supplementary Figure 6: (**A**) Quantitative expression of alterations in expression of tight junction-associated proteins ZO-1, Cldn1, Cldn7 and E-cadherin in normal and oral tumor tissues. (**B**) Quantitative analysis of immunofluorescence micrographs of tumor tissue and adjacent normal tissue of HNSCC patients, stained with FITC-conjugated anti-ZO-1 antibody. (**C**) Graphical representation impact of arecoline on transcription of tight junction-associated genes in HEp-2 cells upon treatment with various concentrations (0, 25, 50, 100, 200, 400, 800 µM) of arecoline for 24 h (upper panel) and 48 h (lower panel). (**D**) Quantitative representation of immunocytochemical analysis of ZO-1 expression in HEp-2 cells after 24 h and 48 h incubation.

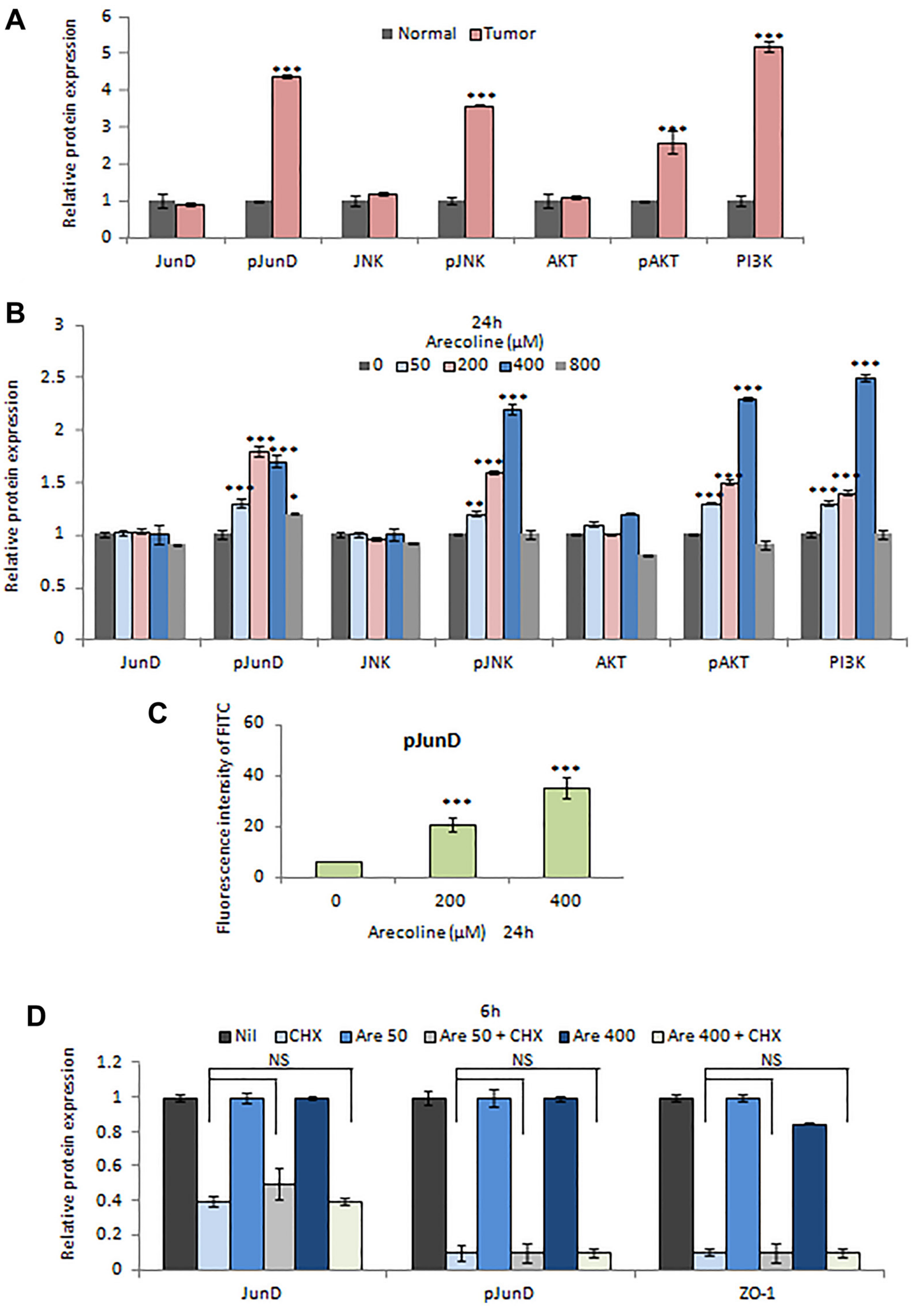

Supplementary Figure 7: (**A**) Quantitative analysis showing expression of MAPK pathway regulator proteins JunD, JNK, AKT along with their phosphorylated forms and PI3K in oral tumor tissues and adjacent normal tissues. (**B**) Quantitative estimation of change in protein expression of MAPK pathway regulators in HEp-2 cells in response to different concentrations (0, 50, 200, 400, 800 µM) of arecoline treatment for 24 h. (**C**) Quantitative representation of immunofluorescence micrographs of HEp-2 cells treated with arecoline for 24 h and stained with anti-pJunD antibody. (**D**) Graphical analysis of differential expression of JunD, pJunD and ZO-1 in HEp-2 cells incubated with 50 µM (+) and 400 µM (++) arecoline in the absence (–) and presence (+) of 100 µg/ml cycloheximide (CHX).

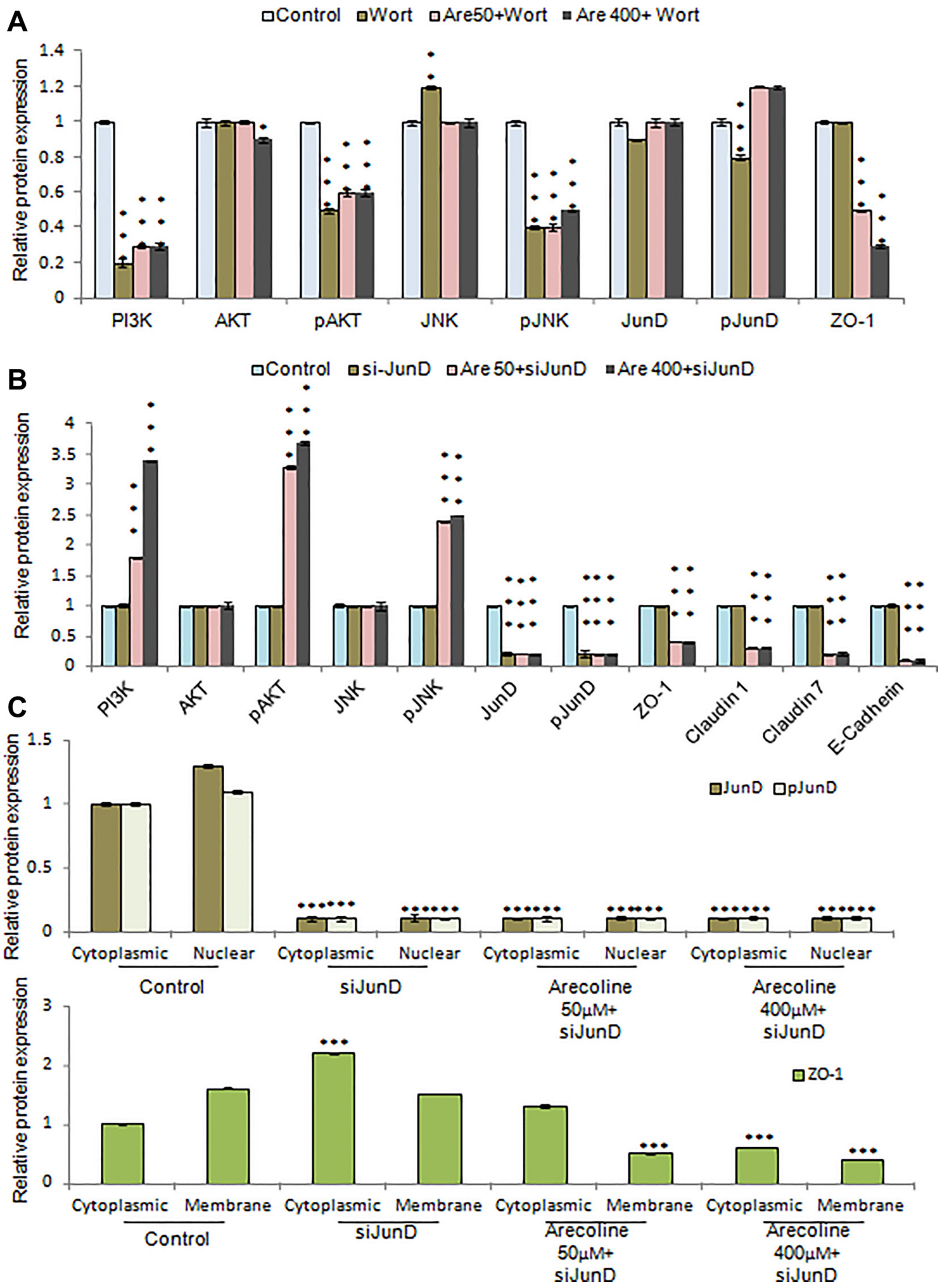

Supplementary Figure 8: (**A**) Quantification graph showing differential protein expression of the MAPK pathway regulators in HEp-2 cells in response to 50 µM (+) or 100 µM (++) arecoline and 100 µM wortmannin as estimated by western blot analysis. (**B**) Quantitative representation of western blot analysis showing effect of JunD silencing on MAPK and tight junction protein components in HEp-2 cells in absence and presence of 50 µM (+) and 100 µM (++) arecoline. (**C**) Graphical representation of effect of JunD silencing followed by arecoline treatment on JunD, pJunD and ZO-1 in the cytoplasmic (C), nuclear (N) and membrane (M) fractions.

Supplementary Table 1: List of antibodies used in the study

Antibody	Make	Catalog no.	Dilution	Secondary Antibody
ZO-1	Santa Cruz	sc10804	1:1000	Mouse α Rabbit
Claudin1	Santa Cruz	sc 17658	1:1000	Mouse α Goat
Claudin7	Santa Cruz	sc 17670	1:1000	Mouse α Goat
E-cadherin	Santa Cruz	sc 7870	1:1000	Mouse α Rabbit
N-cadherin	Santa Cruz	sc 8424	1:1000	Rabbit α Mouse
Slug	Santa Cruz	sc 166476	1:1000	Rabbit α Mouse
Snail	Santa Cruz	sc 271977	1:1000	Rabbit α Mouse
Twist	Santa Cruz	sc 81417	1:1000	Rabbit α Mouse
ALDH1A	Abcam	ab9883	1:1000	Mouse α Goat
SOX2	Abcam	ab 92494	1:1000	Mouse α Rabbit
OCT4	Abcam	ab 27985	1:1000	Mouse α Goat
Nanog	Abcam	ab 80892	1:1000	Mouse α Rabbit
PI3k	Santa Cruz	sc 1637	1:1000	Rabbit α Mouse
AKT	Santa Cruz	sc 5298	1:1000	Rabbit α Mouse
pAKT	Santa Cruz	sc 293125	1:1000	Rabbit α Mouse
JNK	Santa Cruz	sc 7345	1:1000	Rabbit α Mouse
pJNK	Santa Cruz	sc 12882	1:1000	Mouse α Goat
JunD	Santa Cruz	sc 74	1:1000	Mouse α Rabbit
pJunD	Santa Cruz	sc 101726	1:1000	Mouse α Rabbit
β- tubulin	Santa Cruz	sc 5274	1:1000	Rabbit α Mouse
Anti-rabbit	Santa Cruz	sc 2357	1:2000	-
Anti-mouse	Santa Cruz	sc 516102	1:2000	-
Anti-goat	Santa Cruz	sc 2354	1:2000	-

Supplementary Table 2: List of gene specific primers

Name	Sequence
ZO-1 Forward	5′- TGAGGCAGCTCACATAATGC-3′
ZO-1 Reverse	5′-GCTCTCTGCTGGCTTGTTTC -3′
Claudin1 Forward	5′-GATGAGGTGCAGAAGATGAGG -3′
Claudin1 Reverse	5′-AGAAGGCAGAGAGAAGCAGC -3′
CLaudin7 Forward	5′-AATGTACGACTCGGTGCTCG -3′
Claudin7 Reverse	5′- AATCTGATGGCCATACCAGG-3′
E-cadherin Forward	5′- GGCGCCACCTGGAGAGA-3′
E-cadherin Reverse	5′-TGTCGACCGGTGCAATCTT -3′
JunD Forward	5′-ATC GAC ATG GAC ACG CAG-3′
JunD Reverse	5′-GGG TCT TCA CTT TCT CTT CCA G-3′
Snail Forward	5′-GGAAGCCTAACTACAGCGAGCT -3′
Snail Reverse	5′- ACGGTTACGAGTAGACCCT-3′
Slug Forward	5′-CTGGTCAAGAAGCATTTCAACGCC -3′
Slug Reverse	5′-ATGGGTTACCGGAGAGAGGAGAAA -3′
Twist Forward	5′-TCTTACGAGGAGCTGCAGAC -3′
Twist Reverse	5′- CTACCGTTCGACGTCGATA-3′
N-cadherin Forward	5′-GCGTCTGTAGAGGCTTCTGG-3′
N-cadherin Reverse	5′-GCCACTTGCCACTTTTCCTG-3′
NEAT1 Forward	5′-CTTCCTCCCTTTAACTTATCCATTCAC-3′
NEAT1 Reverse	5′-CTCTTCCTCCACCATTACCAACAATAC-3′
MALAT1 Forward	5′-GAATTGCGTCATTTAAAGCCTAGTT-3′
MALAT1 Reverse	5′-GTTTCATCCTACCACTCCCAATTAAT-3′
MEG3 Forward	5′-CTCCCCTTCTAGCGCTCACG-3′
MEG3 Reverse	5′-CTAGCCGCCGTCTATACTACCGGCT-3′
HAR1A Forward	5′-GGCCTGGAAACCCTCTAAAA-3′
HAR1A Reverse	5′-AAACGGGACACACCAGAGTC-3′
XIST1 Forward	5′ GGATGTCAAAAGATCGGCCC3′
XIST1 Reverse	5′ GTCCTCAGGTCTCACATGCT3′
